# Meta-Analysis of Neurochemical Changes Estimated via Magnetic Resonance Spectroscopy in Mild Cognitive Impairment and Alzheimer's Disease

**DOI:** 10.3389/fnagi.2021.738971

**Published:** 2021-10-22

**Authors:** Huanhuan Liu, Dandan Zhang, Huawei Lin, Qi Zhang, Ling Zheng, Yuxin Zheng, Xiaolong Yin, Zuanfang Li, Shengxiang Liang, Saie Huang

**Affiliations:** ^1^National-Local Joint Engineering Research Center of Rehabilitation Medicine Technology, Fujian University of Traditional Chinese Medicine, Fuzhou, China; ^2^Rehabilitation Industry Institute, Fujian University of Traditional Chinese Medicine, Fuzhou, China; ^3^College of Rehabilitation Medicine, Fujian University of Traditional Chinese Medicine, Fuzhou, China; ^4^College of Traditional Chinese Medicine, Fujian University of Traditional Chinese Medicine, Fuzhou, China; ^5^Academy of Integrative Medicine, Fujian University of Traditional Chinese Medicine, Fuzhou, China; ^6^Fujian Key Laboratory of Integrative Medicine on Geriatrics, Fuzhou, China; ^7^Traditional Chinese Medicine Rehabilitation Research Center of State Administration of Traditional Chinese Medicine, Fujian University of Traditional Chinese Medicine, Fuzhou, China; ^8^Department of Neurological Rehabilitation, Fujian University of Traditional Chinese Medicine Subsidiary Rehabilitation Hospital, Fuzhou, China

**Keywords:** magnetic resonance spectroscopy, Alzheimer's disease, mild cognitive impairment, meta-analysis, myo-inositol, N-acetyl aspartate

## Abstract

The changes of neurochemicals in mild cognitive impairment (MCI) and Alzheimer's disease (AD) patients has been observed via magnetic resonance spectroscopy in several studies. However, whether it exists the consistent pattern of changes of neurochemicals in the encephalic region during the progression of MCI to AD were still not clear. The study performed meta-analysis to investigate the patterns of neurochemical changes in the encephalic region in the progress of AD. We searched the PubMed, Embase, Cochrane Library, and Web of Science databases, and finally included 63 studies comprising 1,086 MCI patients, 1,256 AD patients, and 1,907 healthy controls. It showed that during the progression from MCI to AD, N-acetyl aspartate (NAA) decreased continuously in the posterior cingulate (PC) (SMD: −0.42 [95% CI: −0.62 to −0.21], *z* = −3.89, *P* < 0.05), NAA/Cr (creatine) was consistently reduced in PC (SMD: −0.58 [95% CI: −0.86 to −0.30], *z* = −4.06, *P* < 0.05) and hippocampus (SMD: −0.65 [95% CI: −1.11 to −0.12], *z* = −2.44, *P* < 0.05), while myo-inositol (mI) (SMD: 0.44 [95% CI: 0.26–0.61], *z* = 4.97, *P* < 0.05) and mI/Cr (SMD: 0.43 [95% CI: 0.17–0.68], *z* = 3.30, *P* < 0.05) were raised in PC. Furthermore, these results were further verified by a sustained decrease in the NAA/mI of PC (SMD: −0.94 [95% CI: −1.24 to −0.65], *z* = −6.26, *P* < 0.05). Therefore, the levels of NAA and mI were associated with the cognitive decline and might be used as potentially biomarkers to predict the possible progression from MCI to AD.

**Systematic Review Registration:** https://www.crd.york.ac.uk/PROSPERO/, identifier: CRD42020200308.

## Introduction

Alzheimer's disease (AD) is a neurodegenerative disease with age-related progressive cognitive impairment (Scheltens et al., 2016). According to Alzheimer's Disease International, the number of AD patients worldwide was about 50 million in 2018, which would be tripled by 2050 (Scheltens et al., 2021). Early detection and identification of the preclinical AD stage has been thought to be extremely important for slowing down the disease progression of AD. As the prodromal state of AD, there are about 31.5% of mild cognitive impairment (MCI) patients who will be converted to AD within 5 years (Ward et al., 2013). Therefore, exploring the potential biomarkers from MCI to AD is critical for early identification and developing evidence-based interventions of the condition.

The aggregation of amyloid-β (Aβ) in Aβ-pleated and the accumulation of tau in neurofibrillary tangles (NFT) is thought to be the key pathological features of AD (Holtzman et al., 2011). The cerebrospinal fluid (CSF) biomarkers Aβ 42, total tau, and phosphorylated tau are found to be sensitive and common biomarkers that can effectively reflect the typical pathological features of AD (Palmqvist et al., 2016; Sun et al., 2020). The main detection methods of these biomarkers are imaging examination such as ^11^C-labeled Pittsburgh compound-B(PIB)-positron emission tomography (PET) or biological fluids examination, especially from CSF (Mattsson-Carlgren et al., 2020). However, the abnormalities of Aβ 42, T-tau, and P-tau may lag behind cognitive impairment (Jack et al., 2013). Moreover, some changes of biomarkers are found at a stage which the basic neuropathological examination has reached an advanced and irreversible state and needed to be tested and verified by autopsy and histopathology. As a result, it is urgent to identify sensitive and specific biomarkers and detection methods for facilitating early detection and effective treatment of AD.

With the advantages of non-invasiveness, higher sensitivity, and without any radiation, magnetic resonance spectroscopy (MRS) has been widely used to assess the changes of neurochemicals in specific brain tissues in MCI and AD. Increasing evidence suggests a link between the incidence and progression of AD and metabolic dysfunction. Studies have found that neurochemicals, including N-acetyl aspartate (NAA), choline (Cho), creatine (Cr), myo-inositol (mI), and glutamate and glutamine (Glx), have abnormal metabolic changes in the pathological process of AD. NAA is a specific metabolite of the nervous system, which is synthesized by aspartic acid and acetyl-CoA in neuronal mitochondria, and is highly expressed in neuronal mitochondria. It is widely considered as a specific indicator of neuronal activity. Studies have shown that the level of NAA is closely associated with cognitive dysfunction, especially memory impairment (Jessen et al., 2000). Moreover, the autopsy results showed that the level of NAA was decreased in AD patients. Cho signal is related to cell membrane phospholipid metabolism, which mainly reflects the damage of cholinergic neurons. When the cell membrane is destroyed, the level of Cho will show an increasing trend. In addition, Cho has a close relationship with learning, recall, and other cognitive abilities (Khomenko et al., 2019). The level of Cr *in vivo* is relatively stable, and is closely related to energy metabolism, maintaining ATP level in cells, but the content is reduced in the late stage of AD. Myo-inositol has a role in the second messenger cycle and is regarded as a marker of glial cells. Studies have found that mI level increased in the hippocampus in MCI but decreased in the late period of AD (Voevodskaya et al., 2016). Glx is a key amino acid in the brain and studies showed that a decrease of Glx and cognitive impairment always occurred simultaneously (Huang et al., 2017). Therefore, research on the changes of these neurochemicals in the brain may be helpful for the early diagnosis of MCI and AD.

In recent years, several studies have applied MRS to detect the metabolic changes of neurochemicals in the brain of MCI and AD patients to predict the progress of the condition. However, the results were various. The ratio of NAA/mI is often used to distinguish AD from normal people, and the sensitivity was as high as 83% (Kantarci et al., 2000). Interestingly, one study suggested that the NAA/mI in the posterior cingulate (PC) of MCI patients decreased (Mitolo et al., 2019), while another study found that the NAA/mI showed an increase trend in the same brain region (García et al., 2008). Previous studies have found that the ratio of NAA/Cr in the medial temporal lobe (MTL) is increased in AD patients, indicating neuron damage in the brain (Jessen et al., 2009). On the contrary, the level decreased markedly in the MTL region of AD patients in another study (Chao et al., 2005). To investigate whether there would be a consistent pattern of changes of neurochemicals in the encephalic region in the progress of AD, a meta-analysis was conducted. The goal was to identify the changes of abnormal neurochemicals in typical brain regions from MCI to AD.

## Methods

This meta-analysis and systematic review were reported according to the Preferred Reporting Items for Systematic Reviews and Meta-Analyses (PRISMA; Moher et al., 2009) and was registered at International Prospective Register of Systematic Reviews (https://www.crd.york.ac.uk/PROSPERO/) (number CRD42020200308).

### Search Strategy

We searched PubMed, Web of Science, Embase, and Cochrane Library databases from database inception to June 1, 2020. The search strategy was [(“Mild cognitive impairment” OR “Alzheimer's Disease”) AND (“magnetic resonance spectroscopy” OR “MRS” OR “MR Spectroscopy”)]. The search was limited to English language studies only. Regardless of the primary outcome or the type of study, we have considered all possible eligible studies for review.

### Selection Criteria

Studies meeting the following criteria were included: (1) the proton MRS was performed to compare MCI patients, AD patients, and healthy controls. (2) NINCSD-ADRDA criteria were chosen as the diagnostic standard for AD, and criteria used for diagnosis of MCI were clearly reported. (3) At least one single metabolites ratio or concentration in a specific brain region was reported. (4) Specifications for spectrum acquisition were reported.

The exclusion criteria were as follows: (1) studies were published in languages other than English. (2) Original data could not be extracted, or the full text could not be obtained. (3) Duplicate or similar data published research. (4) The subjects were animals. (5) The subjects were taking drugs, had other significant medical conditions or substance abuse that could interfere with cognitive functioning.

### Data Extraction

After applying the inclusion and exclusion criteria, we finally identified 63 articles and extracted the following characteristics for meta-analysis: the interested brain regions and the corresponding metabolites ratios and concentrations, the field strength, repetition time/echo time (TR/TE), and other characteristics which are shown in Table 1. Meanwhile, we also extracted standard deviations (SD) or standard error of mean (SEM) or median, as our main results.

**Table 1:** Characteristics of included studies for the meta-analysis.

	**References**	**Area**	**Field** **(Tesla)**	**Pulse**	**TR/TE (ms)**	**Subject total (HC/AD/MCI)**	**Metabolites**				**MMSE (mean ± SD)**		
							**Ratio**		**Concentration**		**HC**	**AD**	**MCI**
#1	Ackl et al. (2005)	Hippocampus Parietal WM Parietal GM	1.5	PRESS	2,000/70	59 (22/18/19)	NAA/Cr mI/NAA	mI/Cr	/		29.4 ± 0.8	23.5 ± 4.4	29.2 ± 1.1
#2	Azevedo et al. (2008)	Temporal Parietal Occipital	1.5	PRESS	2,000/35	28 (15/13/–)	NAA/Cr Cho/Cr	mI/Cr	NAA Cho	Cr mI	26.53 ± 3.14	16.15 ± 4.02	/
#3	Bai et al. (2015)	Frontal Parietal	3	PRESS	8.2/3.7	30 (15/15/–)	GABA+/Cr	GM/(GM + WM)	/		29.20 ± 0.86	15.87 ± 5.03	/
#4	Block et al. (2002)	Hippocampus Temporal Occipital	1.5	/	2,400/20	56 (22/34/–)	NAA/tCr	Cho/tCr	/		28.6 ± 2.1	20.1 ± 4.5	/
#5	Catani et al. (2001)	PWM	1.5	PRESS	2,000/40	36 (11/14/11)	NAA/Cr Cho/Cr	mI/Cr	/		29.8 ± 0.4	20.3 ± 2.5	27 ± 2.5
#6	Catani et al. (2002)	PWM	1.5	PRESS	2,000/40	10 (10/10/–)	NAA/Cr Cho/Cr	mI/Cr	/		29 ± 0.5	20 ± 2	/
#7	Chantal et al. (2002)	MTLs PTCs FCs	1.5	PRESS	1,200/51	28 (14/14/–)	NAA/H_2_O Cho/H_2_O	Cr/H2O mI/H2O	//		29.3 ± 0.9	22.9 ± 4	/
#8	Chao et al. (2005)	MTL Frontal GM Parietal GM	1.5	PRESS	1,800/135	48 (24/24/–)	NAA/Cr		NAA		29 ± 0.8	17.4 ± 6.7	/
#9	Chao et al. (2010)	PC	1.5	STEAM	1,800/25	22 (9/–/13)	NAA/Cr NAA/mI	mI/Cr	/		29.6 ± 0.6	/	27 ± 2.2
#10	de Souza et al. (2011)	PC	1.5	PRESS	1,200 or 1,500/31	68 (33/25/10)	NAA/Cr Cho/Cr	mI/Cr mI/NAA	/		27.7 ± 2.09	20.45 ± 4.59	25.7 ± 2.49
#11	Delli et al. (2015)	Thalamus	3	PRESS	2,000/39	29 (13/16/–)	NAA/tCr tCho/tCr	tCr/H2O	/		28.3 ± 1.3	17.7 ± 4.5	/
#12	Ding et al. (2008)	PC	1.5	PRESS	1,500/35	40 (20/20/–)	NAA/Cr Cho/Cr	mI/Cr	/		28.3 ± 1.0	11.8 ± 3.8	/
#13	Ernst et al. (1997)	Frontal Temporo-parietal	1.5	PRESS	3,000/35	23 (11/12/–)	NAA/Cr Cho/Cr	mI/Cr	NAA Cr		/	/	/
#14	Fayed et al. (2011)	PC	1.5	PRESS	2,000/35	124 (26/30/68)	NAA/Cr Cho/Cr mI/Cr	Glu/Cr Glx/Cr	NAA Cho mI	Glu Glx	/	/	/
#15	Fayed et al. (2014)	PC	2.5	PRESS	2,000/36	295 (193/36/66)	NAA/Cr Cho/Cr mI/Cr	Glu/Cr Glx/Cr	NAA Cho mI	Glu Glx	/	/	/
#16	Fernández et al. (2005)	Temporo-parietal	1.5	PRESS	3,000/96	20 (10/10/–)	NAA/Cr mI/Cr	NAA/Cho mI/NAA	NAA Cho	mI Cr	34.2 ± 1.03	18.6 ± 4.8	/
#17	Foy et al. (2011)	Hippocampus	1.5	PRESS	1,500/35	98 (39/38/21)	/		NAA Cho	Mi Cr + Pcr	28.8 ± 2.3	23 ± 4	27.1 ± 1.5
#18	Franczak et al. (2007)	Hippocampus	0.5	PRESS	1,500/41	10 (5/–/5)	NAA/Cr mI/Cr mI/NAA	Cho/Cr Glx/Cr Glx/NAA	NAA Cho mI	Cr Glx	≥29	/	≥24
#19	Frederick et al. (2004)	Temporal	1.5	PRESS	2,000/135	29 (14/15/–)	NAA/Cr mI/Cr	Cho/Cr mI/NAA	/		29.1 ± 0.9	17.1 ± 5.5	/
#20	García et al. (2008)	PC	1.5	PRESS	1,500/35 1,500/144	44 (34/–/10)	NAA/Cr mI/Cr	Cho/Cr	/		22.35 ± 1.54	/	22 ± 1.63
#21	Graff-Radford et al. (2014)	PC Occipital Frontal	1.5	PRESS	2,000/30	183 (148/35/–)	NAA/Cr mI/Cr NAA/Cho	NAA/mI Cho/Cr	/		/	/	/
#22	Griffith et al. (2010)	PC	3	PRESS	2,000/32	71 (42/–/29)	NAA/Cr mI/Cr	Cho/Cr	/		29.43 ± 1.04	/	28 ± 1.44
#23	Guo et al. (2016)	AC PC	3	PRESS	1,500/35	44 (16/15/13)	NAA/Cr mI/Cr	NAA/ mI Cho/Cr	/		29.5 ± 0.21	20.5 ± 2.42	26.1 ± 1.32
#24	Herminghaus et al. (2003)	Parietal GM Parietal WM Frontal WM Frontal GM Temporal	1.5	STEAM	2,000/68	75 (27/48/–)	tNAA/tCr TMA/tCr	Ins/Cr Glx/tCr	/		/	/	/
#25	Huang et al. (2017)	Hippocampus AC	1.5	PRESS	1,500/21	53 (15/17/21)	Glx/tCr GABA+/Cr	NAA/Cr	/		29.07 ± 0.96	16.47 ± 5.33	26.45 ± 2.28
#26	Jessen et al. (2005)	MTL	1.5	PRESS	2,700/120	56 (23/33/–)	NAA/Cr		NAA Cho	Cr	/	20.6 ± 4.5	/
#27	Jessen et al. (2000)	MTL Central region	1.5	PRESS	2,000/272	38 (18/20/–)	NAA/Cr	Cho/Cr	NAA/Cho	Cr	29.1 ± 0.9	19.1 ± 6.1	/
#28	Jessen et al. (2009)	MTL	1.5	/	2,000/272 2,000/30	213 (45/98/70)	NAA/Cr	mI/NAA	NAA Cho	Cr mI	28.9 ± 1	24.7 ± 3.7	26.9 ± 4
#31	Kantarci et al. (2000)	Temporal Occipital PC	1.5	PRESS	2,000/135 2,000/30	105 (63/21/21)	NAA/Cr mI/Cr	Cho/Cr	/		28.6 ± 1.3	18.4 ± 5.9	26.6 ± 2.8
#29	Kantarci et al. (2002)	PC	1.5	PRESS	2,000/30	107 (61/–/24)	NAA/Cr mI/Cr	NAA/mI	/		29	20	28
#30	Kantarci et al. (2007)	PC	1.5	PRESS	2,000/30	194 (85/60/49)	NAA/Cr mI/Cr	Cho/Cr	/		29	23	27
#32	Lazeyras et al. (1998)	CGM SGM WM	1.5	STEAM	1,500/20	29 (14/15/–)	/		Cho Cr	NAA mI	/	14-26	/
#33	Li et al. (2010)	Frontal Temporal Parietal	1.5	/	1,500/30	68 (34/–/34)	NAA/Cr		NAA	Cr	/	/	/
#34	Lim et al. (2012)	AC PC	3	PRESS	2,000/9.177	78 (23/ 36 /19)	NAA/Cr	mI/Cr	/		27 ± 4.4	18.8 ± 5.3	25.1 ± 4.0
#35	Liu et al. (2013)	Hippocampus	1.5	PRESS	1,378/110	39 (18/–/21)	NAA/Cr mI/Cr Cho/Cr	NAA/Cho NAA/mI			26.17 ± 0.86	/	25.1 ± 2.5
#36	Liu et al. (2014)	PC Occipital WM Frontal WM Dorsal Thalamus Inferior precuneus	2.5	PRESS	1,500/35	57 (32/–/25)	NAA/Cr mI/Cr Cho/Cr	NAA/Cho NAA/mI	Cho Cr	NAA mI	28.08 ± 1.38	/	26.13 ± 1.78
#37	MacKay et al. (1996)	Anterior and posterior mesial cortex	2	/	3,000/30 3,000/80	32 (18/14/–)	NAA/Cr Cho/Cr	NAA/Cho	/		29 ± 0.8	14 ± 9	/
#38	Mandal et al. (2015)	Hippocampus Frontal	3	PRESS	2,500/120	64 (21/21/22) (Hippocampus) 66 (28/19/19) (Frontal)	/		GSH		28.7 ± 1.1 (Hippocampus) 29 ± 1.2 (Frontal)	18.4 ± 4.1 (Hippocampus) 18.4 ± 4.7 (Frontal)	25.5 ± 4.1 (Hippocampus) 27.4 ± 1.7 (Frontal)
#39	Marjańska et al. (2019)	PC Occipital	7	PRESS	5,000/8	49 (33/16/–)	/		NAA Cho Cr mI Glu GSH	Gln Asc Asp NAAG GABA sIns	/	19 ± 2	/
#40	Metastasio et al. (2006)	PWM	1.5	PRESS	2,000/40	54 (29/–/25)	NAA/Cr Cho/Cr	mI/Cr	/		29.2 ± 0.9	/	26.96 ± 2.16
#41	Mitolo et al. (2019)	PC	1.5	PRESS	4,000/35	81 (18/25/38)	NAA/mI		/		/	/	/
#42	Modrego et al. (2011)	Parietal Occipital	1.5	PRESS	2,000/35	106 (35/–/71)	NAA/Cr Cho/Cr	mI/Cr NAA/mI	NAA		/	//	
#43	Oeltzschner et al. (2019)	AC PC	7	/	3,000/14 3,000/15	26 (13/–/13)	/		mI NAA GABA	Glu GSH NAAG	28.7 ± 1.2	/	27.5 ± 1.7
#44	Olson et al. (2008)	PC	1.5	/	3,000/20	71 (24/–/47)	NAA/Cr NAA/Cho Cho/Cr	mI/Cr NAA/mI Glx/Cr	Cho Cr NAA	mI Glx	29 ± 1.3	/	27.7 ± 1.9
#45	Parnetti et al. (1997)	Temporal GM Frontal WM	1.5	/	2,600/35	20 (7/13)	/		Cho Cr	NAA mI	>26	14.7 ± 5.4	/
#46	Pilatus et al. (2009)	Parietal GM Parietal WM	1.5	PRESS	3,000/22	27 (12/–/15)	/		Cho Cr NAA	mI Glx	29.8 ± 0.39	/	26.4 ± 2.6
#47	Rami et al. (2007)	PC Temporal Temporo-parietal	1.5	PRESS	1,500/35	89 (27/35/27)	NAA/Cr Cho/Cr	mI/Cr	NAA Cr	Cho mI	27.5 ± 1	21.8 ± 3.8	25.1 ± 2.1
#48	Riese et al. (2015)	PC	2	PRESS	1,800/68	36 (21/–/15)	/		GABA Glx	NAA	29.7 ± 0.6	/	28.6 ± 1.2
#49	Schuff et al., 1998	Mesial cortex centrum semiovale	1.5	PRESS	3,000/80	50 (22/28/–)	/		NAA Cho	Cr	29.3 ± 1	19.1 ± 6.9	/
#50	Schuff et al. (2002)	MTL Frontal Parietal Hippocampus	1.5	PRESS	1,800/135	110 (54/56/–)	/		NAA		29.1 ± 0.8	19 ± 6.7	/
#51	Seo et al. (2012)	PC Hippocampus ERC Occipital WM	3	PRESS	2,000/40	24 (11/–/13)	NAA/Cr	Cho/Cr	/		28.5 ± 1.1	/	25.2 ± 2.3
#52	Shiino et al. (2012)	PC Hippocampus	1.5	PRESS	2,000/30	144 (45/99/–)	NAA/Cr Cho/Cr mI/Cr	Glx/Cr mI/NAA	NAA Glx Cho	mI Cr	29.1 ± 1.2	19.7 ± 3.4	/
#53	Siger et al. (2009)	Frontal Parietal	1.5	/	2,500/20	47 (16/17/14)	/		NAA	mI	29.5 ± 0.9	21.4 ± 5.4	27.6 ± 1.5
#54	Targosz-Gajniak et al. (2013)	PC Hippocampus Parietal	1.5	PRESS	1,500/35	76 (35/–/41)	NAA/Cr Cho/Cr mI/Cr	Glx/Cr NAA/Cho	/		/	/	/
#55	Wang et al. (2009)	Hippocampus PC	3	PRESS	1,700/30	48 (16/16/16)	NAA/Cr Cho/Cr	mI/Cr mI/NAA	/		28.13 ± 1.25	15.63 ± 7.25	26.5 ± 1.51
#56	Wang et al. (2012)	Hippocampus PC	3	PRESS	1,500/35	40 (56/47/32)	NAA/Cr Cho/Cr	mI/Cr NAA/mI	/		26.5 ± 3.5	13.8 ± 5.4	23.9 ± 3.8
#57	Watanabe et al. (2010)	Hippocampus Occipital PC ApPoDeepWM	1.5	PRESS	2,000/30	169 (52/70/47)	/		NAA mI	Cho Cr	29 ± 1.4	20.8 ± 3.6	27.2 ± 1.8
#58	Yang et al. (2012)	PC PWM Inferior precuneus Dorsal thalamus Lentiform nucleus	1.5	PRESS	1,500/35	29 (15/–/14)	NAA/Cr mI/Cr	Cho/Cr NAA/mI	NAA mI	Cho Cr	28.11 ± 1.23	/	25.79 ± 1.06
#59	Zeydan et al. (2017)	PC	3	LASER	2,300/2.98	46 (32/–/14)	Glu/mI		NAA mI Cho	Cr Glu	28	/	26
#60	Zhang et al. (2009)	Hippocampus Temporo-parietal	1.5	/	2,000/25	40 (13/13/14)	NAA/Cr	mI/Cr	/		/	/	/
#61	Zhu et al. (2006)	Parietal GM Parietal WM Front GM Front WM	1.5	/	/	36 (22/14/–)	NAA/Cr mI/Cr	NAA/mI	NAA	mI	29.7 ± 0.5	20 ± 6.7	/
#62	Zhu et al. (2015)	Hippocampus Basal ganglia Frontal	3	PRESS	1,500/30	62 (34/–/28)	NAA/Cr mI/Cr	Cho/Cr	/		28.35 ± 1.3	7/±26	11 ± 1.17
#63	Zimny et al. (2011)	PC	1.5	PRESS	1,500/35	68 (15/30/23)	NAA/Cr mI/Cr Cho/Cr	mI/NAA mI/Cho	/		29.8 ± 0.4	18 ± 5.4	27.4 ± 2.4

*AC, anterior cingulate cortex; AD, Alzheimer's disease; ApPoDeepWM, anterior and posterior deep white matter; Asc, ascorbate; Asp, aspartate; CGM, cortical gray matter; Cho, choline; Cr, creatine; DT, dorsal thalamus; ERC, entorhinal cortex; GABA, γ-aminobutyric acid; Gln, glutamine; Glu, glutamine; Glx, glutamate + glutamine; GM, gray matter; GSH, glutathione; HC, healthy controls; Ins, inositol; LA, left anterior periventricular and deep white matter; LN, lentiform nucleus; LP, left posterior periventricular and deep white matter; MCI, mild cognitive impairment; mI, myo-inositol; MMSE, mini mental state examination; MTL, medial temporal lobe; NAA, N-acetyl aspartate; NAAG, N-acetylaspartylglutamate; PC, posterior cingulate cortex; PCr, phosphocreatine; PR, inferior precuneus; PRESS, point resolved spectroscopy sequence; PTC, parietotemporal cortices; PWM, paratrigonal white matter; RA, right anterior periventricular and deep white matter; RP, right posterior periventricular and deep white matter; SD, standard deviation; SGM, subcortical gray matter; sIns, scyllo-inositol; tCr, creatine and creatine phosphate; TE, echo time (ms); TMA, trimethylamines; TR, repetition time (ms),WM, white matter*.

### Statistical Analyses

Stata 16.0 (Stata Corp) software was used to perform all statistical analysis. The sample size, mean value, and SD were used to generate the effective sizes, and when the mean ± SEM or median was provided, we converted it into mean ± SD for meta-analysis. Then, we calculated the standardized mean difference (SMD) and 95% CI and drew a forest map to compare the relationship between the metabolites' concentrations or metabolites' ratios between the healthy control group and AD patients, the healthy control group and MCI patients, and AD patients and MCI patients. We used the *Q*-test and *I*^2^ index to evaluate heterogeneity. The statistical significance of the *Q*-test was set as *P* < 0.1, and heterogeneity was assessed by *I*^2^ index, with 25, 50, and 75%, indicating that the heterogeneity was low, medium, and high (Higgins et al., 2003). For the statistical model, we first chose fixed effects model with the method of inverse-variance for analysis. If the heterogeneity of the results is greater, we used the random effect model with the method of Dersimonian and Laird for analysis. All statistical significances were set at *P* < 0.05 and *P* < 0.1 was regarded as a trend.

## Results

### Description of Studies

**Figure 1:**
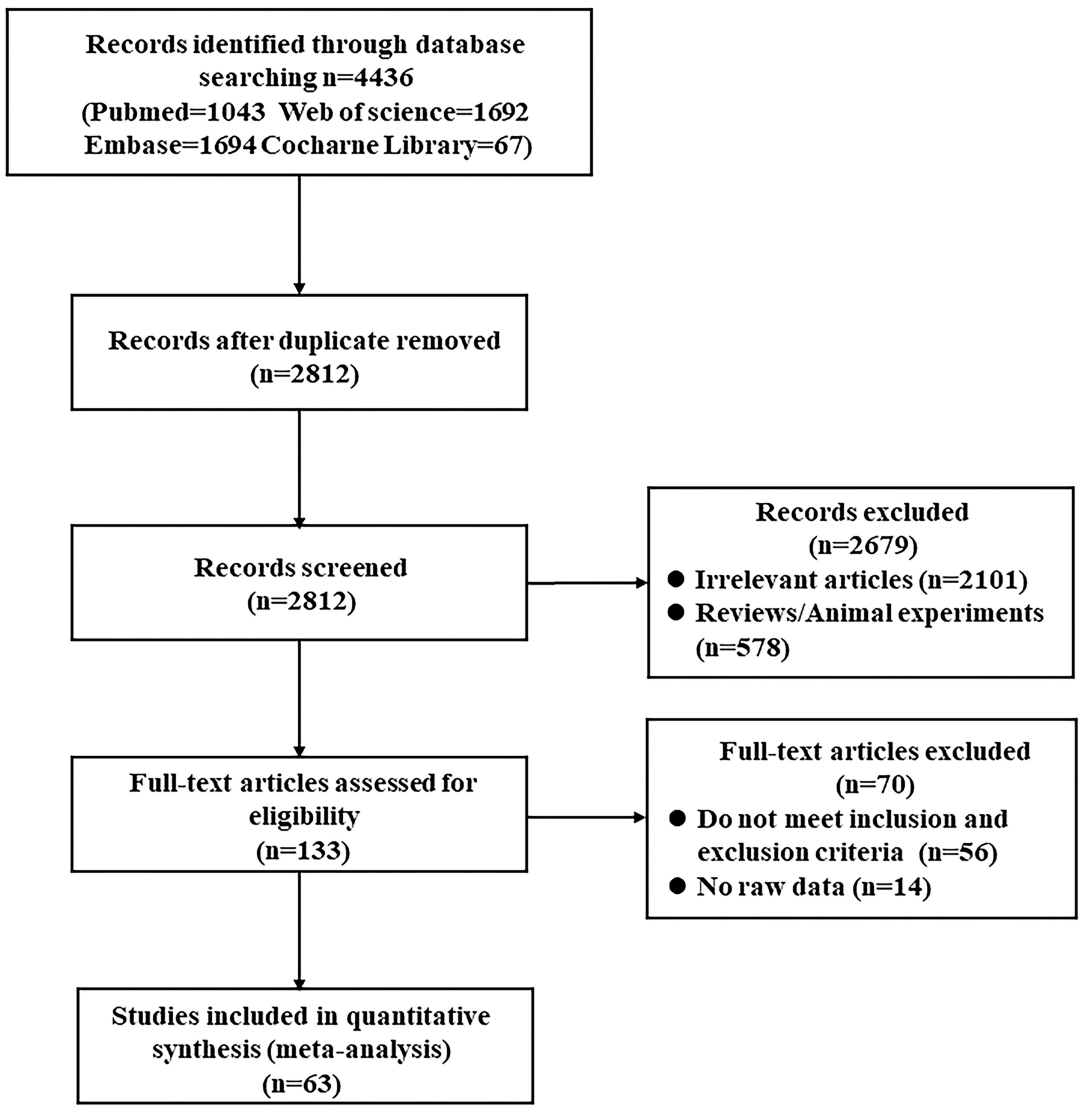
Flowchart for study screening process.

We identified 4,436 articles from database searching and 2,812 remained after removal of duplicates. Then, 133 full-text articles were assessed for eligibility. Finally, according to the inclusion and exclusion criteria, 63 studies were included in the systematic review and meta-analysis (MacKay et al., 1996; Ernst et al., 1997; Parnetti et al., 1997; Lazeyras et al., 1998; Schuff et al., 1998, 2002; Jessen et al., 2000, 2005, 2009; Kantarci et al., 2000, 2002, 2007; Catani et al., 2001, 2002; Block et al., 2002; Chantal et al., 2002; Huang et al., 2002; Herminghaus et al., 2003; Frederick et al., 2004; Ackl et al., 2005; Chao et al., 2005, 2010; Fernández et al., 2005; Metastasio et al., 2006; Zhu et al., 2006, 2015; Franczak et al., 2007; Rami et al., 2007; Azevedo et al., 2008; Ding et al., 2008; García et al., 2008; Pilatus et al., 2009; Siger et al., 2009; Wang et al., 2009, 2012; Zhang et al., 2009; Griffith et al., 2010; Li et al., 2010; Liu et al., 2010, 2013; Watanabe et al., 2010; de Souza et al., 2011; Fayed et al., 2011, 2014; Foy et al., 2011; Modrego et al., 2011; Zimny et al., 2011; Lim et al., 2012; Seo et al., 2012; Shiino et al., 2012; Yang et al., 2012; Targosz-Gajniak et al., 2013; Graff-Radford et al., 2014; Bai et al., 2015; Delli et al., 2015; Mandal et al., 2015; Riese et al., 2015; Guo et al., 2016; Zeydan et al., 2017; Marjańska et al., 2019; Mitolo et al., 2019; Oeltzschner et al., 2019; Figure 1).

The meta-analysis comprised of a total of 3,271 subjects, with 1,086 MCI patients, 1,256 AD patients, and 1,907 healthy controls. The following regions were investigated: hippocampus (including MTL), PC, temporal lobe, occipital regions, paratrigonal white matter area, temporo-parietal lobe, parietal lobe, frontal lobe (gray and white matter area), and anterior cingulate. The key characteristics of the studies are shown in Table 1 among which 6 studies were classified as high quality and 57 studies were classified as medium quality (Table 2).

**Table 2:** The Newcastle-Ottawa Scale (NOS) for the quality assessment of studies.

**References**	**Selection**				**Comparability**		**Exposure**				**Total**
	**S1**	**S2**	**S3**	**S4**	**C1**	**C2**	**E1a**	**E1b**	**E2**	**E3**	
Ackl et al. (2005)	^*^	–	–	^*^	^*^	^*^	–	–	^*^	–	5
Azevedo et al. (2008)	^*^	^*^	^*^	^*^	^*^	^*^	–	–	^*^	–	7
Bai et al. (2015)	^*^	–	–	^*^	^*^	^*^	–	–	^*^	–	5
Block et al. (2002)	^*^	–	–	^*^	^*^	^*^	–	–	^*^	–	5
Catani et al. (2001)	^*^	–	–	^*^	^*^	^*^	–	–	^*^	–	5
Catani et al. (2002)	^*^	–	–	^*^	^*^	^*^	–	–	^*^	–	5
Chantal et al. (2002)	^*^	–	–	^*^	^*^	^*^	–	–	^*^	–	5
Chao et al. (2005)	^*^	^*^	^*^	^*^	^*^	^*^	–	–	^*^	–	7
Chao et al. (2010)	^*^	^*^	^*^	^*^	^*^	–	–	–	^*^	–	6
de Souza et al. (2011)	^*^	^*^	–	^*^	^*^	^*^	–	–	^*^	–	6
Delli et al. (2015)	^*^	–	–	^*^	^*^	^*^	–	–	^*^	–	5
Ding et al. (2008)	^*^	–	–	^*^	^*^	^*^	–	–	^*^	–	5
Ernst et al. (1997)	^*^	–	–	^*^	^*^	^*^	–	–	^*^	–	5
Fayed et al. (2011)	^*^	–	–	^*^	^*^	^*^	–	–	^*^	^*^	6
Fayed et al. (2014)	^*^	–	–	^*^	^*^	^*^	–	–	^*^	–	5
Fernández et al. (2005)	^*^	^*^	–	^*^	^*^	^*^	–	–	^*^	–	6
Foy et al. (2011)	^*^	^*^	–	^*^	^*^	^*^	–	–	^*^	–	6
Franczak et al. (2007)	^*^	–	–	^*^	^*^	^*^	–	–	^*^	–	5
Frederick et al. (2004)	^*^	^*^	^*^	^*^	^*^	^*^	–	–	^*^	–	7
García et al. (2008)	^*^	–	^*^	^*^	^*^	^*^	–	–	^*^	^*^	7
Graff-Radford et al. (2014)	^*^	^*^	–	^*^	^*^	^*^	–	–	^*^	–	6
Griffith et al. (2010)	^*^	^*^	^*^	^*^	^*^	^*^	–	–	^*^	–	7
Guo et al. (2016)	^*^	^*^	–	^*^	^*^	^*^	–	–	^*^		6
Herminghaus et al. (2003)	^*^	^*^	–	^*^	^*^	^*^	–	–	^*^	–	6
Huang et al. (2017)	^*^	^*^	^*^	^*^	^*^	–	–	–	^*^	–	6
Jessen et al. (2000)	^*^	–	–	^*^	^*^	^*^	–	–	^*^		5
Jessen et al. (2005)	^*^	^*^	–	^*^	^*^	^*^	–	–	^*^	–	6
Jessen et al. (2009)	^*^	–	–	^*^	^*^	^*^	–	–	^*^	–	5
Kantarci et al. (2000)	^*^	–	–	^*^	^*^	^*^	–	–	^*^	–	5
Kantarci et al. (2002)	^*^	^*^	–	^*^	^*^	^*^	–	–	^*^	–	6
Kantarci et al. (2007)	^*^	^*^	–	^*^	^*^	^*^	–	–	^*^	^*^	7
Lazeyras et al. (1998)	^*^	–	–	^*^	^*^	^*^	–	–	^*^	–	5
Li et al. (2010)	^*^	–	–	^*^	^*^	^*^	–	–	^*^	–	5
Lim et al. (2012)	^*^	–	–	^*^	^*^	^*^	–	–	^*^	–	5
Liu et al. (2013)	^*^	–	–	^*^	^*^	^*^	–	–	^*^	–	5
Liu et al. (2014)	^*^	–	–	^*^	^*^	^*^	–	–	^*^	–	5
MacKay et al. (1996)	^*^	^*^	–	^*^	^*^	^*^	–	–	^*^	–	6
Mandal et al. (2015)	^*^	^*^	^*^	^*^	^*^	^*^	–	–	^*^	–	7
Marjańska et al. (2019)	^*^	–	–	^*^	^*^	^*^	–	–	^*^	–	5
Metastasio et al. (2006)	^*^	–	–	^*^	^*^	^*^	–	–	^*^		5
Mitolo et al. (2019)	^*^	–	–	^*^	^*^	^*^	–	–	^*^	^*^	6
Modrego et al. (2011)	^*^	–	–	^*^	^*^	^*^	–	–	^*^	–	5
Oeltzschner et al. (2019)	^*^	^*^	^*^	^*^	^*^	^*^	–	–	^*^	–	7
Olson et al. (2008)	^*^	–	^*^	^*^	^*^	^*^	–	–	^*^	^*^	7
Parnetti et al. (1997)	^*^	^*^	^*^	^*^	^*^	–	–	–	^*^	–	6
Pilatus et al. (2009)	^*^	–	^*^	^*^	^*^	^*^	–	–	^*^	^*^	7
Rami et al. (2007)	^*^	^*^	–	^*^	^*^	^*^	–	–	^*^	–	6
Riese et al. (2015)	^*^	–	–	^*^	^*^	^*^	–	–	^*^	–	5
Schuff et al. (1998)	^*^	–	^*^	^*^	^*^	^*^	–	–	^*^	–	6
Schuff et al. (2002)	^*^	–	^*^	^*^	^*^	^*^	–	–	^*^	–	6
Seo et al. (2012)	^*^	–	–	^*^	^*^	^*^	–	–	^*^	–	5
Shiino et al. (2012)	^*^	^*^	–	^*^	^*^	^*^	–	–	^*^	–	6
Siger et al. (2009)	^*^	–	^*^	^*^	^*^	^*^	–	–	^*^	–	6
Targosz-Gajniak et al. (2013)	^*^	^*^	^*^	^*^	^*^	^*^	–	–	^*^	^*^	8
Wang et al. (2009)	^*^	–	^*^	^*^	^*^	^*^	–	–	^*^	–	6
Wang et al. (2012)	^*^	–	^*^	^*^	^*^	^*^	–	–	^*^	–	6
Watanabe et al. (2010)	^*^	^*^	–	^*^	^*^	^*^	–	–	^*^	–	6
Yang et al. (2012)	^*^	–	–	^*^	^*^	^*^	–	–	^*^	–	5
Zeydan et al. (2017)	^*^	^*^	–	^*^	^*^	^*^	–	–	^*^	–	6
Zhang et al. (2009)	^*^	–	–	^*^	^*^	^*^	–	–	^*^	–	5
Zhu et al. (2006)	^*^	–	^*^	^*^	^*^	^*^	–	–	^*^	–	6
Zhu et al. (2015)	^*^	–	–	^*^	^*^	^*^	–	–	^*^	–	5
Zimny et al. (2011)	^*^	–	–	^*^	^*^	^*^	–	–	^*^	–	5

* *means that this study awarded one score on this question*.

### Meta-Analysis of Hippocampus

Nineteen studies (Jessen et al., 2000, 2005, 2009; Block et al., 2002; Schuff et al., 2002; Ackl et al., 2005; Chao et al., 2005; Franczak et al., 2007; Wang et al., 2009, 2012; Zhang et al., 2009; Watanabe et al., 2010; Foy et al., 2011; Seo et al., 2012; Shiino et al., 2012; Liu et al., 2013; Targosz-Gajniak et al., 2013; Zhu et al., 2015; Huang et al., 2017) investigated the ratios of metabolites in hippocampus from 358 MCI patients, 890 AD patients, and 787 healthy control subjects. Specifically, 12 studies (Ackl et al., 2005; Franczak et al., 2007; Wang et al., 2009, 2012; Zhang et al., 2009; Watanabe et al., 2010; Foy et al., 2011; Seo et al., 2012; Liu et al., 2013; Targosz-Gajniak et al., 2013; Zhu et al., 2015; Huang et al., 2017) performed a comparison of the changes between 358 MCI patients and 425 healthy control subjects, 14 studies (Jessen et al., 2000, 2005, 2009; Block et al., 2002; Schuff et al., 2002; Ackl et al., 2005; Chao et al., 2005; Wang et al., 2009, 2012; Zhang et al., 2009; Watanabe et al., 2010; Foy et al., 2011; Shiino et al., 2012; Huang et al., 2017) compared the differences in metabolites between 890 AD patients and 679 healthy control subjects, and 5 studies (Ackl et al., 2005; Wang et al., 2009, 2012; Zhang et al., 2009; Huang et al., 2017) were conducted to observe the differences of metabolites in 155 AD patients and 130 MCI patients. Moreover, there were another two articles (Modrego et al., 2011; Seo et al., 2012) longitudinally tracking the metabolite differences in the hippocampus between MCI-converter and MCI-stable patients.

#### Metabolite Ratios

**Figure 2:**
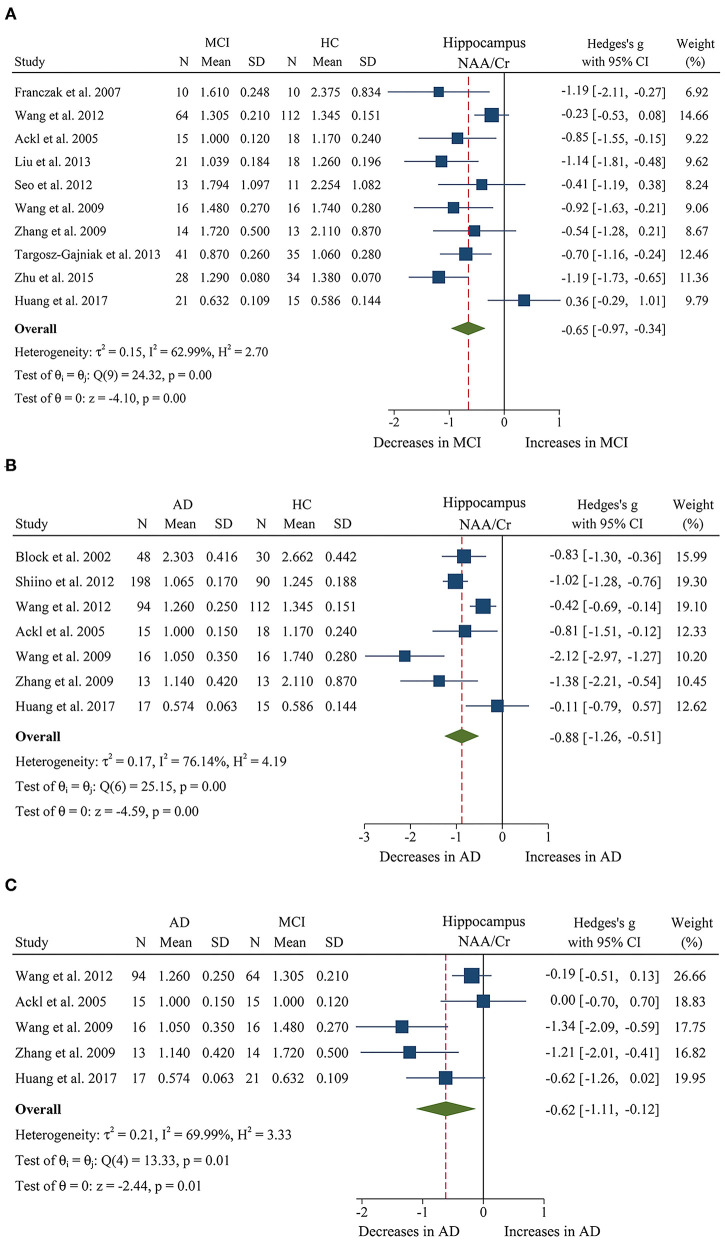
Forest plots show the change of the ratio of NAA/Cr in the hippocampus between MCI, AD patients, and HC subjects. **(A)** Data include 525 individuals from 10 studies for meta-analysis of NAA/Cr levels between MCI and HC. **(B)** Data include 695 individuals from 7 studies for meta-analysis of NAA/Cr levels between AD and HC. **(C)** Data include 285 individuals from 5 studies for meta-analysis of NAA/Cr levels between AD and MCI.

We compared the ratios of five metabolites, extracting data from 243 MCI patients and 282 healthy control subjects in 10 studies (Ackl et al., 2005; Franczak et al., 2007; Wang et al., 2009, 2012; Zhang et al., 2009; Seo et al., 2012; Liu et al., 2013; Targosz-Gajniak et al., 2013; Zhu et al., 2015; Huang et al., 2017). The results showed that four metabolites' ratios (NAA/Cr, Cho/Cr, mI/Cr, and mI/NAA) were significantly different in MCI and healthy control subjects, but there was no significant difference in Glx/Cr (SMD: −0.76 [95% CI: −1.81 to 0.28], *z* = −1.44, *P* > 0.1, Supplementary Table 1). Among them, NAA/Cr (SMD: −0.65 [95% CI: −0.97 to −0.34], *z* = −4.10, *P* < 0.05, Figures 2A, **9**) and Cho/Cr (SMD: −0.20 [95% CI: −0.39 to −0.01], *z* = −2.09, *P* < 0.05, Supplementary Figure 1A and **Figure 9**) were significantly decreased in the hippocampus of MCI patients, while mI/Cr (SMD: 0.52 [95% CI: 0.20–0.83], *z* = 3.24, *P* < 0.05, Supplementary Figure 1B and **Figure 9**) and mI/NAA (SMD: 1.58 [95% CI: 0.71–2.45], *z* = 3.55, *P* < 0.05, Supplementary Figure 1C and **Figure 9**) were significantly increased.

In addition, 11 studies (Jessen et al., 2000, 2005, 2009; Block et al., 2002; Ackl et al., 2005; Chao et al., 2005; Wang et al., 2009, 2012; Zhang et al., 2009; Shiino et al., 2012; Huang et al., 2017) were combined to compare the ratios of NAA/Cr, Cho/Cr, mI/Cr, and mI/NAA in 600 AD patients and 428 healthy control subjects. The results showed that four metabolites' ratios (NAA/Cr, Cho/Cr, mI/Cr, and mI/NAA) were significantly different in AD and healthy control subjects. For the comparisons between AD and controls, NAA/Cr (SMD: −0.88 [95% CI: −1.25 to −0.51], *z* = −4.59, *P* < 0.05, Figures 2B, **9**) and Cho/Cr (SMD: −0.23 [95% CI: −0.39 to −0.06], *z* = −2.67, *P* < 0.05, Supplementary Figure 2A and **Figure 9**) were significantly decreased in the hippocampus of AD patients, while mI/Cr (SMD: 0.93 [95% CI: 0.76–1.11], *z* = 10.40, *P* < 0.05, Supplementary Figure 2B and **Figure 9**) and mI/NAA (SMD: 1.98 [95% CI: 0.81–3.15], *z* = 3.31, *P* < 0.05, Supplementary Figure 2C and **Figure 9**) showed a significant increase. Moreover, 4 studies of MTL were eligible for inclusion, comprising 199 AD patients and 134 healthy controls, all AD compared to controls NAA/Cr (SMD: −0.48 [95% CI: −0.93 to −0.03], *z* = −2.07, *P* < 0.05, Supplementary Figure 3A and **Figure 9**) were decreased.

Next, we performed a meta-analysis to compare the ratios of NAA/Cr, between 155 AD patients and 130 MCI patients (Ackl et al., 2005; Wang et al., 2009, 2012; Zhang et al., 2009; Huang et al., 2017). The results demonstrated significant difference. NAA/Cr (SMD: −0.62 [95% CI: −1.11 to −0.12], *z* = −2.44, *P* < 0.05, Figures 2C, **9**) in the MCI group were significantly higher than that in the AD patients. Four studies (Ackl et al., 2005; Wang et al., 2009, 2012; Zhang et al., 2009) investigated the mI/Cr from the hippocampus of 137 AD patients and 109 MCI patients, and the results showed that the mI/Cr ratio in the AD patients (SMD: 0.25 [95% CI: −0.01 to 0.50], *z* = 1.92, *P* = 0.055, Supplementary Table 1) were increased compared to the MCI patients.

Moreover, studies (Modrego et al., 2011; Seo et al., 2012) longitudinally tracked the metabolite differences in the hippocampus between MCI-converter and MCI-stable patients. Compared with MCI-stable patients, a downward trend in Cho/Cr (SMD: −0.47 [95%CI: −0.94 to 0.01], *z* = −1.91, *P* = 0.06, Supplementary Table 1 and **Figure 9**) was observed in MCI-converter patients. Meanwhile, the analysis on NAA/Cr (SMD: −0.17 [95% CI: −0.65 to 0.30], *z* = −0.72, *P* > 0.05, Supplementary Table 1 and **Figure 9**) revealed no significant difference between the two groups.

Above all, according to the results of meta-analysis of AD and MCI, the ratios of NAA/Cr and Cho/Cr are both decreased in AD and MCI patients. Notably, the decrease was more obvious in AD patients. In addition, mI/Cr and mI/NAA ratios were seen to increase faster in AD patients, compared to subjects who converted to MCI and cognitively normal elderly.

#### Metabolite Concentrations

Of the eligible studies, 4 studies (Franczak et al., 2007; Watanabe et al., 2010; Foy et al., 2011; Liu et al., 2013) investigated metabolite concentrations. These studies comprised 146 MCI patients and 171 healthy controls. The analysis showed that four metabolites' concentrations (NAA, Cr, Cho, and mI) were significantly different in MCI and healthy control subjects, and no significant difference in mI concentration (SMD: 0.23 [95% CI: −0.19 to 0.65], *z* = 1.08, *P* > 0.1, Supplementary Table 1). Among them, NAA (SMD: −1.01 [95% CI: −1.25 to −0.78], *z* = −8.45, *P* < 0.05, Supplementary Figure 1D and **Figure 9**), Cr (SMD: −1.35 [95% CI: −2.50 to −0.20], *z* = −2.30, *P* < 0.05, Supplementary Figure 1F and **Figure 9**), and Cho (SMD: −0.55 [95% CI: −0.78 to −0.33], *z* = −4.80, *P* < 0.05, Supplementary Figure 1E and **Figure 9**) were low heterogeneity and remarkably decreased in the hippocampus of MCI patients.

Then, 4 studies (Schuff et al., 2002; Watanabe et al., 2010; Foy et al., 2011; Shiino et al., 2012) were extracted to compare the metabolite concentrations in 488 AD patients and 341 healthy control subjects. The analysis revealed that NAA, Cr, and Cho concentrations were significantly different in AD and healthy control subjects, while there was no difference of the concentration of mI between AD and healthy controls (SMD: 0.46 [95% CI: −0.11 to 1.03], *z* = 1.57, *P* > 0.1, Supplementary Table 1). For the comparisons between AD and controls, NAA (SMD: −1.17 [95% CI: −1.61 to −0.74], *z* = −0.53, *P* < 0.05, Supplementary Figure 2D and **Figure 9**), Cho (SMD: −0.58 [95% CI: −0.75 to −0.42], *z* = −6.82, *P* < 0.05, Supplementary Figure 2E and **Figure 9**), and Cr (SMD: −0.44 [95% CI: −0.71 to −0.16], *z* = −3.12, *P* < 0.05, Supplementary Figure 2F and **Figure 9**) concentrations were significantly decreased in the hippocampus of AD patients with statistically high heterogeneity. In addition, 4 studies (Jessen et al., 2000, 2005, 2009; Chao et al., 2005) of MTL were eligible for inclusion, comprising 288 AD patients and 221 healthy controls, all AD compared to controls NAA (SMD: −0.89 [95% CI: −1.08 to −0.7], *z* = −9.40, *P* < 0.05, Supplementary Figure 3B and **Figure 9**) were decreased.

In conclusion, based on the analysis of AD and MCI, 3 metabolites' concentrations (NAA, Cr, and Cho) were found to be lower in AD patients as compared to MCI patients and healthy control subjects.

### Meta-Analysis of Posterior Cingulate

A total of 29 studies (Kantarci et al., 2000, 2002, 2007; Chao et al., 2005; Rami et al., 2007; García et al., 2008; Olson et al., 2008; Wang et al., 2009, 2012; Griffith et al., 2010; Watanabe et al., 2010; de Souza et al., 2011; Fayed et al., 2011, 2014; Zimny et al., 2011; Lim et al., 2012; Seo et al., 2012; Shiino et al., 2012; Yang et al., 2012; Targosz-Gajniak et al., 2013; Graff-Radford et al., 2014; Liu et al., 2014; Riese et al., 2015; Guo et al., 2016; Zeydan et al., 2017; Marjańska et al., 2019; Mitolo et al., 2019; Oeltzschner et al., 2019) investigated the ratio of metabolites in posterior cingulate with a sample size of 770 MCI patients, 585 AD patients, and 1,378 healthy controls. To be specific, 25 studies (Kantarci et al., 2000, 2002, 2007; Chao et al., 2005; Rami et al., 2007; García et al., 2008; Olson et al., 2008; Wang et al., 2009, 2012; Griffith et al., 2010; Watanabe et al., 2010; de Souza et al., 2011; Fayed et al., 2011, 2014; Zimny et al., 2011; Lim et al., 2012; Seo et al., 2012; Yang et al., 2012; Targosz-Gajniak et al., 2013; Liu et al., 2014; Riese et al., 2015; Guo et al., 2016; Zeydan et al., 2017; Mitolo et al., 2019; Oeltzschner et al., 2019) compared the differences in metabolites between 770 MCI patients and 1,132 healthy control subjects, 16 studies (Kantarci et al., 2007; Rami et al., 2007; Ding et al., 2008; Wang et al., 2009, 2012; Watanabe et al., 2010; de Souza et al., 2011; Fayed et al., 2011, 2014; Zimny et al., 2011; Lim et al., 2012; Shiino et al., 2012; Graff-Radford et al., 2014; Guo et al., 2016; Marjańska et al., 2019; Mitolo et al., 2019) compared the differences in metabolites between 610 AD patients and 822 healthy control subjects, and 12 studies (Kantarci et al., 2007; Rami et al., 2007; Wang et al., 2009, 2012; Watanabe et al., 2010; de Souza et al., 2011; Fayed et al., 2011, 2014; Zimny et al., 2011; Lim et al., 2012; Guo et al., 2016; Mitolo et al., 2019) made a comparison between 440 AD patients and 421 MCI patients. Moreover, there were another two studies (Kantarci et al., 2007; Seo et al., 2012) longitudinally tracking the metabolite differences between MCI-converter and MCI-stable patients.

#### Metabolite Ratios

**Figure 3:**
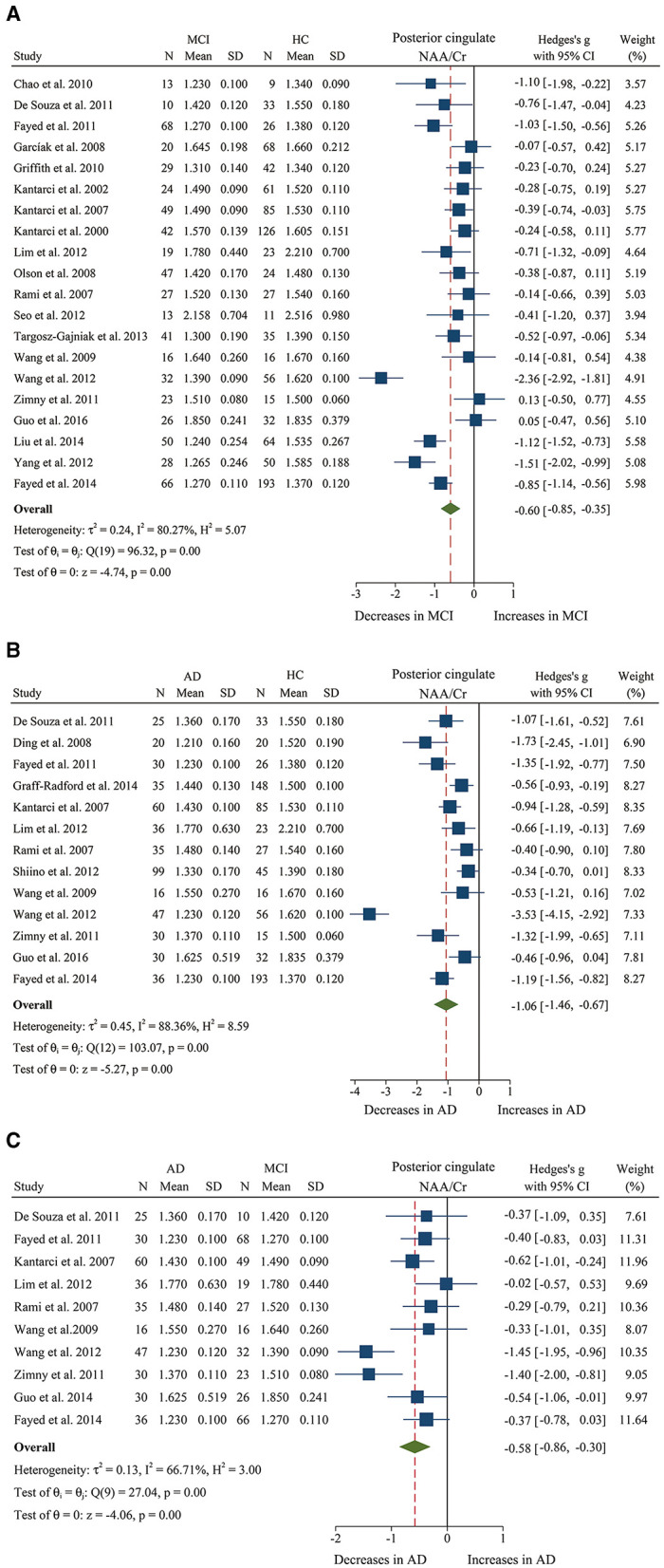
Forest plots show the change of the ratio of NAA/Cr in the posterior cingulate between MCI, AD patients, and HC subjects. **(A)** Data include 1639 individuals from 20 studies for meta-analysis of NAA/Cr levels between MCI and HC. **(B)** Data include 1218 individuals from 13 studies for meta-analysis of NAA/Cr levels between AD and HC. **(C)** Data include 681 individuals from 5 studies for meta-analysis of NAA/Cr levels between AD and MCI.

**Figure 4:**
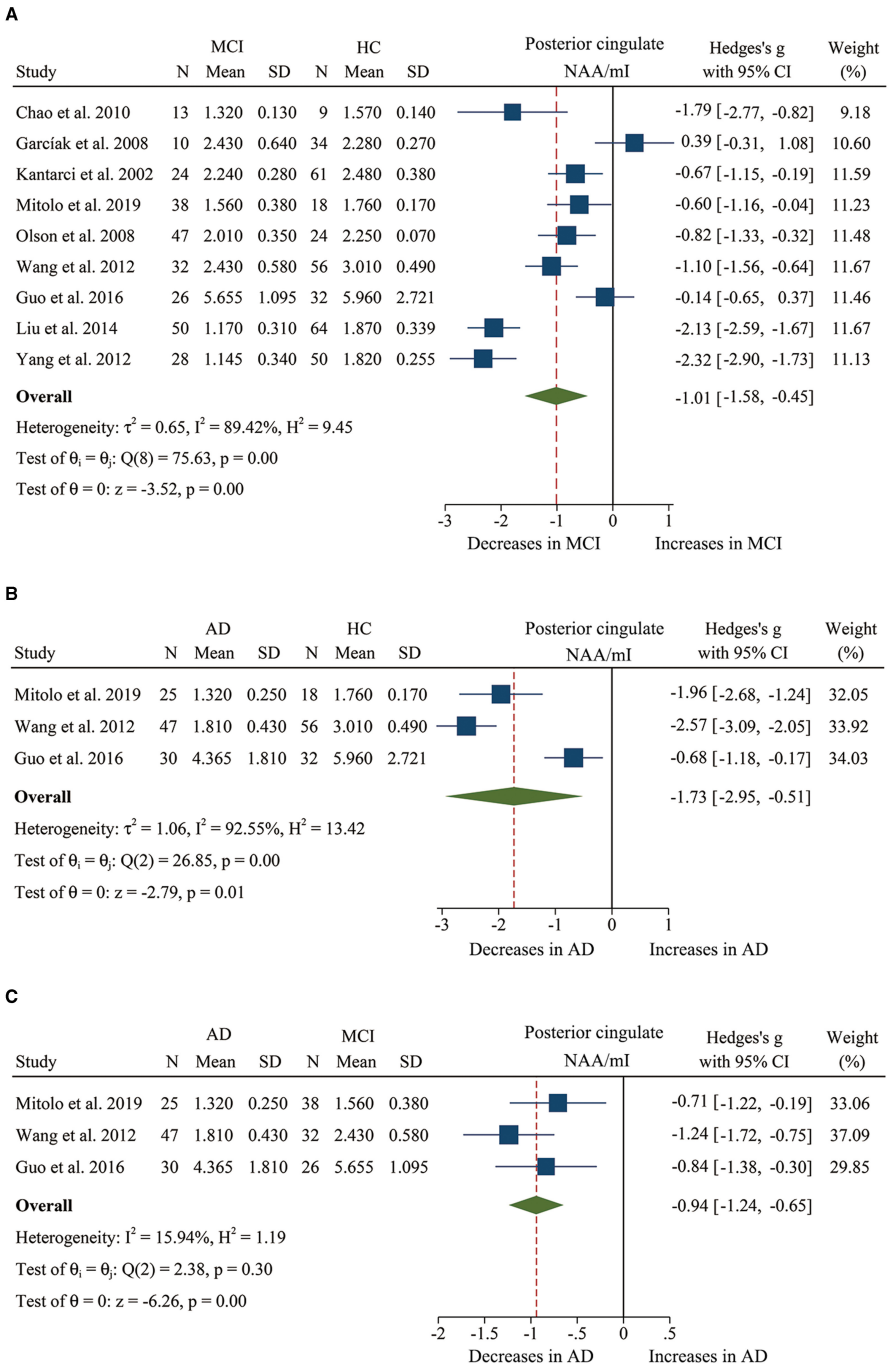
Forest plots show the change of the ratio of NAA/mI in the posterior cingulate between MCI, AD patients, and HC subjects. **(A)** Data include 616 individuals from 9 studies for meta-analysis of NAA/mI levels between MCI and HC. **(B)** Data include 208 individuals from 3 studies for meta-analysis of NAA/mI levels between AD and HC. **(C)** Data include 198 individuals from 3 studies for meta-analysis of NAA/mI levels between AD and MCI.

**Figure 5:**
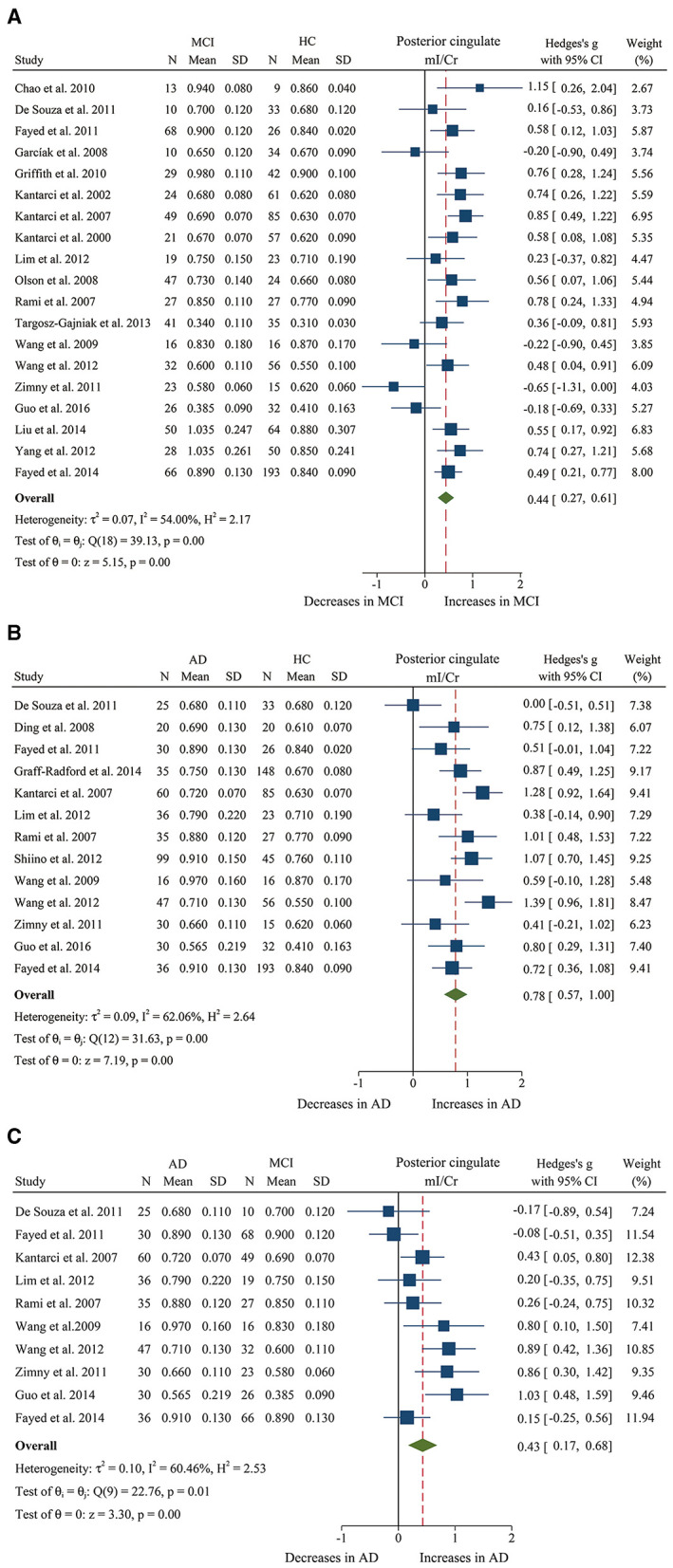
Forest plots show the change of the ratio of mI/Cr in the posterior cingulate between MCI, AD patients, and HC subjects. **(A)** Data include 1481 individuals from 19 studies for meta-analysis of mI/Cr levels between MCI and HC. **(B)** Data include 1,218 individuals from 13 studies for meta-analysis of mI/Cr levels between AD and HC. **(C)** Data include 681 individuals from 5 studies for meta-analysis of mI/Cr levels between AD and MCI.

**Figure 6:**
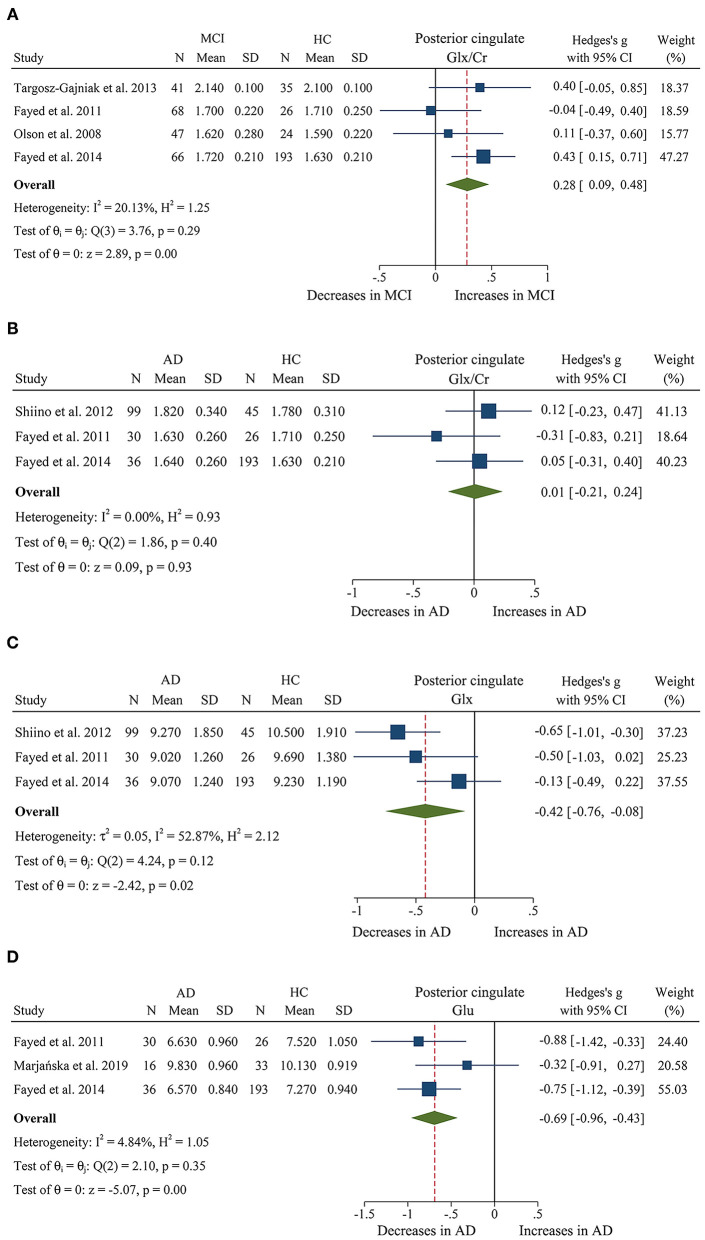
Forest plots show the change of the levels of Glx/Cr, Glx, and Glu in the posterior cingulate between MCI, AD patients, and HC subjects. **(A)** Data include 500 individuals from 4 studies for meta-analysis of Glx/Cr levels between MCI and HC. **(B)** Data include 429 individuals from 3 studies for meta-analysis of Glx/Cr levels between AD and HC. **(C)** Data include 429 individuals from 3 studies for meta-analysis of Glx levels between AD and HC. **(D)** Data include 334 individuals from 3 studies for meta-analysis of Glu levels between AD and HC.

We finally identified 21 studies (Kantarci et al., 2000, 2002, 2007; Rami et al., 2007; García et al., 2008; Olson et al., 2008; Wang et al., 2009, 2012; Chao et al., 2010; Griffith et al., 2010; de Souza et al., 2011; Fayed et al., 2011, 2014; Zimny et al., 2011; Lim et al., 2012; Seo et al., 2012; Yang et al., 2012; Targosz-Gajniak et al., 2013; Guo et al., 2016; Mitolo et al., 2019) with a total sample size of 1,695 (681 MCI patients and 1,014 healthy controls) comparing the metabolite ratio in the posterior cingulate. The results showed that NAA/Cr (SMD: −0.60 [95% CI: −0.85 to −0.35], *z* = −4.74, *P* < 0.05, Figures 3A, **9**) and NAA/mI (SMD: −1.01 [95% CI: −1.58 to −0.45], *z* = −3.52, *P* < 0.05, Figures 4A, **9**) were significantly decreased in MCI patients than in healthy controls, while mI/Cr (SMD: 0.44 [95% CI: 0.27–0.61], *z* = 5.15, *P* < 0.05, Figures 5A, **9**) and Glx/Cr (SMD: 0.28 [95% CI: 0.09–0.48], *z* = 2.89, *P* < 0.05, Figures 6A, **9**) were significantly increased. There was no significant difference in the ratio of mI/NAA (SMD: −0.02 [95% CI: −0.79 to 0.82], *z* = 0.04, *P* > 0.1, Supplementary Table 1). Besides, Cho/Cr (SMD: 0.34 [95% CI: −0.00 to 0.69], *z* = 1.96, *P* > 0.05, Supplementary Table 1) has an uptrend in the posterior cingulate of MCI patients. On the contrary, there was a downward trend in NAA/Cho (SMD: −0.35 [95% CI: −0.72 to 0.03], *z* = −1.80, *P* > 0.05, Supplementary Table 1).

Additionally, 14 studies (Kantarci et al., 2007; Rami et al., 2007; Ding et al., 2008; Wang et al., 2009, 2012; de Souza et al., 2011; Fayed et al., 2011, 2014; Zimny et al., 2011; Lim et al., 2012; Shiino et al., 2012; Graff-Radford et al., 2014; Guo et al., 2016; Mitolo et al., 2019) were analyzed to compare the metabolite ratios in 600 AD patients and 428 healthy control subjects. The results showed that five metabolites' ratios (NAA/Cr, mI/Cr, Cho/Cr, mI/NAA, NAA/mI, and Glx/Cr) were significantly different in AD and healthy control subjects. For the comparisons between AD and controls, NAA/Cr (SMD: −1.06 [95% CI: −1.46 to −0.67], *z* = −5.27, *P* < 0.05, Figures 3B, **9**) and NAA/mI (SMD: −1.73 [95% CI: −2.95 to −0.51], *z* = −2.79, *P* < 0.05, Figures 4B, **9**) were significantly decreased in the posterior cingulate of AD patients, while mI/Cr (SMD: 0.78 [95% CI: 0.57–1.00], *z* = 7.19, *P* < 0.05, Figures 5B, **9**), Glx/Cr (SMD: 0.01 [95% CI: −0.21 to 0.24], *z* = 0.09, *P* < 0.05, Figure 6B), mI/NAA (SMD:1.01 [95% CI: 0.75–1.26], *z* = 7.64, *P* < 0.05, Supplementary Figure 4A and **Figure 9**), and Cho/Cr (SMD:0.35 [95% CI: 0.11–0.59], *z* = 2.85, *P* < 0.05, Supplementary Figure 4B and **Figure 9**) were remarkably increased.

Next, we performed a meta-analysis to compare the ratios in the posterior cingulate, comprising 370 AD patients and 374 MCI patients (Kantarci et al., 2007; Rami et al., 2007; Wang et al., 2009, 2012; de Souza et al., 2011; Fayed et al., 2011, 2014; Zimny et al., 2011; Lim et al., 2012; Guo et al., 2016; Mitolo et al., 2019). The results demonstrated that NAA/Cr (SMD: −0.58 [95% CI: −0.86 to −0.30], *z* = −4.06, *P* < 0.05, Figures 3C, **9**) and NAA/mI (SMD: −0.94 [95% CI: −1.24 to −0.65], *z* = −6.26, *P* < 0.05, Figures 4C, **9**) were significantly higher in the MCI group than that in the AD patients. Meanwhile, the analysis revealed a remarkable increase in mI/Cr (SMD: 0.43 [95% CI: 0.17–0.68], *z* = 3.03, *P* < 0.05, Figures 5C, **9**) and mI/NAA (SMD: 0.92 [95% CI: 0.31–1.53], *z* = 2.97, *P* < 0.05, Supplementary Figure 4C and **Figure 9**) with a high heterogeneity.

Two studies were extracted to compare the ratios in 25 MCI-converter patients and 37 MCI-stable patients (Kantarci et al., 2007; Seo et al., 2012). The results revealed that there was no difference in NAA/Cr (SMD: 0.17 [95% CI: −0.33 to −0.67], *z* = 0.68, *P* > 0.1, Supplementary Table 1) and Cho/Cr (SMD: 0.11 [95% CI: −0.39 to 0.61], *z* = −0.44, *P* > 0.1, Supplementary Table 1).

Taken together, these results suggest that the ratios of NAA/Cr and NAA/mI were reduced in AD patients as compared to MCI patients and healthy controls. However, in the posterior cingulate, mI/NAA and Glx/Cr decreased remarkably compared to that of AD patients.

#### Metabolite Concentrations

**Figure 7:**
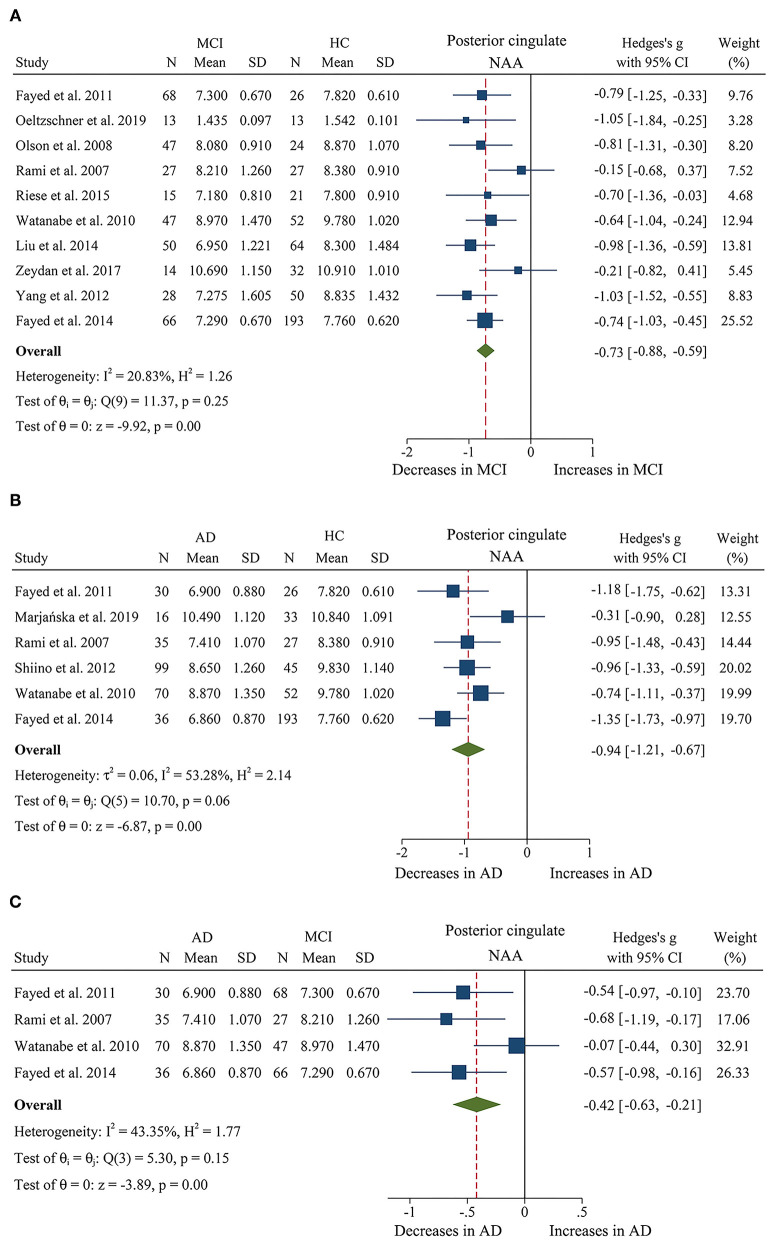
Forest plots show the change of NAA in the posterior cingulate during the development from healthy people to AD. **(A)** Data include 877 individuals from 10 studies for meta-analysis of NAA levels between MCI and HC. **(B)** Data include 662 individuals from 6 studies for meta-analysis NAA levels between AD and HC. **(C)** Data include 379 individuals from 4 studies for meta-analysis NAA levels between AD and MCI.

We compared the concentrations of metabolites, extracting data from 10 studies with a sample size of 375 MCI patients and 502 healthy control subjects (Rami et al., 2007; Olson et al., 2008; Watanabe et al., 2010; Fayed et al., 2011, 2014; Yang et al., 2012; Liu et al., 2014; Riese et al., 2015; Zeydan et al., 2017; Oeltzschner et al., 2019). The analyses showed that NAA was significantly decreased in the posterior cingulate of MCI patients (SMD: −0.73 [95% CI: −0.88 to −0.59], *z* = −9.92, *P* < 0.05, Figures 7A, **9**), while mI was significantly increased (SMD: 0.54 [95% CI: 0.39–0.69], *z* = 7.24, *P* < 0.05, Supplementary Figure 4D and **Figure 9**). There was no significant difference in the concentrations of Cr (SMD: −0.17 [95% CI: −0.44 to 0.10], *z* = −1.24, *P* > 0.1), Cho (SMD: 0.12 [95% CI: −0.03 to 0.27], *z* = 1.60, *P* > 0.1), and Glx (SMD: −0.08 [95% CI: −0.62 to 0.46], *z* = −0.46, *P* > 0.1). Besides, three studies were included to investigate Glu concentration, and the analysis revealed a downward trend with a high heterogeneity (SMD: −0.44 [95% CI: −0.94 to 0.06], *z* = −1.74, *P* = 0.08).

Then, the metabolite concentrations were compared in 6 studies with 286 AD patients and 376 healthy controls (Rami et al., 2007; Watanabe et al., 2010; Fayed et al., 2011, 2014; Shiino et al., 2012; Marjańska et al., 2019). The results demonstrated that NAA (SMD: −0.94 [95% CI: −1.21 to −0.67], *z* = −6.87, *P* < 0.05, Figures 7B, **9**), Glu (SMD: −0.69 [95% CI: −0.96 to −0.43], *z* = −5.07, *P* < 0.05, Figures 6D, **9**), and Glx (SMD: −0.42 [95% CI: −0.76 to −0.08], *z* = −2.42, *P* < 0.05, Figures 6C, **9**) were significantly higher in healthy controls than that in the AD patients, while mI (SMD: 0.44 [95% CI: 0.26–0.61], *z* = 4.97, *P* < 0.05, Supplementary Figure 4E and **Figure 9**) was lower than that in the AD patients. Besides, 4 studies were included to investigate Cr concentration and the analysis revealed a downward trend with a high heterogeneity (SMD: −0.37 [95% CI: −0.80 to 0.05], *z* = −1.71, *P* > 0.05, Supplementary Table 1 and **Figure 9**). Six studies (Rami et al., 2007; Watanabe et al., 2010; Fayed et al., 2011, 2014; Shiino et al., 2012; Marjańska et al., 2019) were included to investigate Cho concentration and the analysis manifested an upward trend with a medium heterogeneity (SMD: 0.23 [95% CI: −0.02 to 0.48], *z* = 1.81, *P* > 0.05, Supplementary Table 1 and **Figure 9**).

Next, we compared the concentrations in the posterior cingulate, with 171 AD patients and 208 MCI patients (Rami et al., 2007; Watanabe et al., 2010; Fayed et al., 2011, 2014). The results demonstrated that NAA was significantly decreased in the AD patients (SMD: −0.42 [95% CI: −0.62 to −0.21], *z* = −3.89, *P* < 0.05, Figures 7C, **9**), while there was no difference in mI (SMD: −0.07 [95% CI: −0.28 to 0.13], *z* = −0.69, *P* > 0.1, Supplementary Table 1) and Cho (SMD: −0.05 [95% CI: −0.57 to 0.48], *z* = −0.17, *P* > 0.1, Supplementary Table 1).

Briefly, according to the results of meta-analysis of AD and MCI, the concentration of NAA was decreased in AD and MCI patients, especially in AD patients. In addition, mI concentration was seen to increase faster in AD patients, compared to subjects who converted to MCI and cognitively normal elderly.

### Meta-Analysis of Temporal Lobe

There were 7 studies (Kantarci et al., 2000; Block et al., 2002; Herminghaus et al., 2003; Frederick et al., 2004; Rami et al., 2007; Azevedo et al., 2008; Li et al., 2010) investigating the ratio of metabolites in the temporal lobe and included 82 MCI patients, 157 AD patients, and 207 healthy controls. Of these studies, 3 (Kantarci et al., 2000; Rami et al., 2007; Li et al., 2010) compared the differences in metabolites between 82 MCI patients and 124 healthy control subjects, and 5 (Block et al., 2002; Herminghaus et al., 2003; Frederick et al., 2004; Rami et al., 2007; Azevedo et al., 2008) compared the differences between 157 AD patients and 110 healthy control subjects.

#### Metabolite Ratios

First, we performed a meta-analysis to compare the ratios of NAA/Cr in the temporal lobe, comprising 82 MCI patients and 124 healthy controls (Kantarci et al., 2000; Rami et al., 2007; Li et al., 2010). The analysis showed that there was no significant difference in NAA/Cr between the two groups (SMD: −0.12 [95% CI: −0.40 to 0.17], *z* = −0.81, *P* > 0.1, Supplementary Table 1).

When comparing AD with controls, 5 studies (Block et al., 2002; Herminghaus et al., 2003; Frederick et al., 2004; Rami et al., 2007; Azevedo et al., 2008) were included for meta-analysis. The results showed that the ratio of NAA/Cr was significantly different between the two groups, and there was a difference in the ratio of Cho/Cr and mI/Cr. The ratio of NAA/Cr (Block et al., 2002; Herminghaus et al., 2003; Frederick et al., 2004; Rami et al., 2007; Azevedo et al., 2008) was remarkably decreased in the AD patients with high heterogeneity (SMD: −0.68 [95% CI: −1.24 to −0.12], *z* = −2.40, *P* < 0.05, Supplementary Figure 4F and **Figure 9**). Meanwhile, Cho/Cr (Block et al., 2002; Frederick et al., 2004; Rami et al., 2007; Azevedo et al., 2008) has a downward trend in the temporal lobe of AD patients (SMD: −0.27 [95% CI: −0.57 to 0.01], *z* = −1.87, *P* > 0.05, Supplementary Table 1). On the contrary, there was an uptrend in mI/Cr (SMD: 0.35 [95% CI: −0.01 to 0.71], *z* = 1.91, *P* > 0.05, Supplementary Table 1).

### Meta-Analysis of the Parietal Lobe

Eight studies (Herminghaus et al., 2003; Ackl et al., 2005; Chao et al., 2005; Zhu et al., 2006; Siger et al., 2009; Li et al., 2010; Modrego et al., 2011; Targosz-Gajniak et al., 2013) with a total sample size of 639 (162 AD patients, 187 MCI patients, and 290 healthy controls) were included for meta-analysis to investigate the ratio of metabolites in the parietal lobe. Specifically, 3 studies (Herminghaus et al., 2003; Ackl et al., 2005; Zhu et al., 2006) compared the differences in metabolites between 80 AD patients and 71 healthy control subjects in parietal WM, and 5 studies (Herminghaus et al., 2003; Ackl et al., 2005; Chao et al., 2005; Zhu et al., 2006; Siger et al., 2009) compared the differences in metabolites between 162 AD patients and 151 healthy control subjects in parietal GM. Moreover, there were 2 studies (Modrego et al., 2005, 2011) longitudinally tracking the metabolite differences between MCI-converter and MCI-stable patients.

#### Metabolite Ratios

We finally identified 3 studies with a total sample size of 326 (187 MCI patients and 139 healthy controls) to compare the ratio of NAA/Cr in the parietal lobe. The analysis revealed that there was no significant difference observed between the two groups (SMD: 0.02 [95% CI: −0.20 to 0.24], *z* = 0.16, *P* > 0.1, Supplementary Table 1).

Next, 3 studies (Herminghaus et al., 2003; Ackl et al., 2005; Zhu et al., 2006) were included to compare the ratio of NAA/Cr in parietal WM, comprising 80 AD patients and 71 healthy controls. The analysis revealed a significant decrease in the AD patients with high significant heterogeneity (SMD: −1.16 [95% CI: −1.72 to −0.60], *z* = −4.06, *P* < 0.05, Supplementary Figure 5A and **Figure 9**) in parietal WM. A meta-analysis of 3 studies (Herminghaus et al., 2003; Ackl et al., 2005; Zhu et al., 2006) limited to the parietal GM lobe showed a remarkable decrease in NAA/Cr in the ADs patients (SMD: −1.10 [95% CI: −2.02 to −0.70], *z* = −2.33, *P* < 0.05, Supplementary Figure 5B and **Figure 9**).

**Figure 8:**
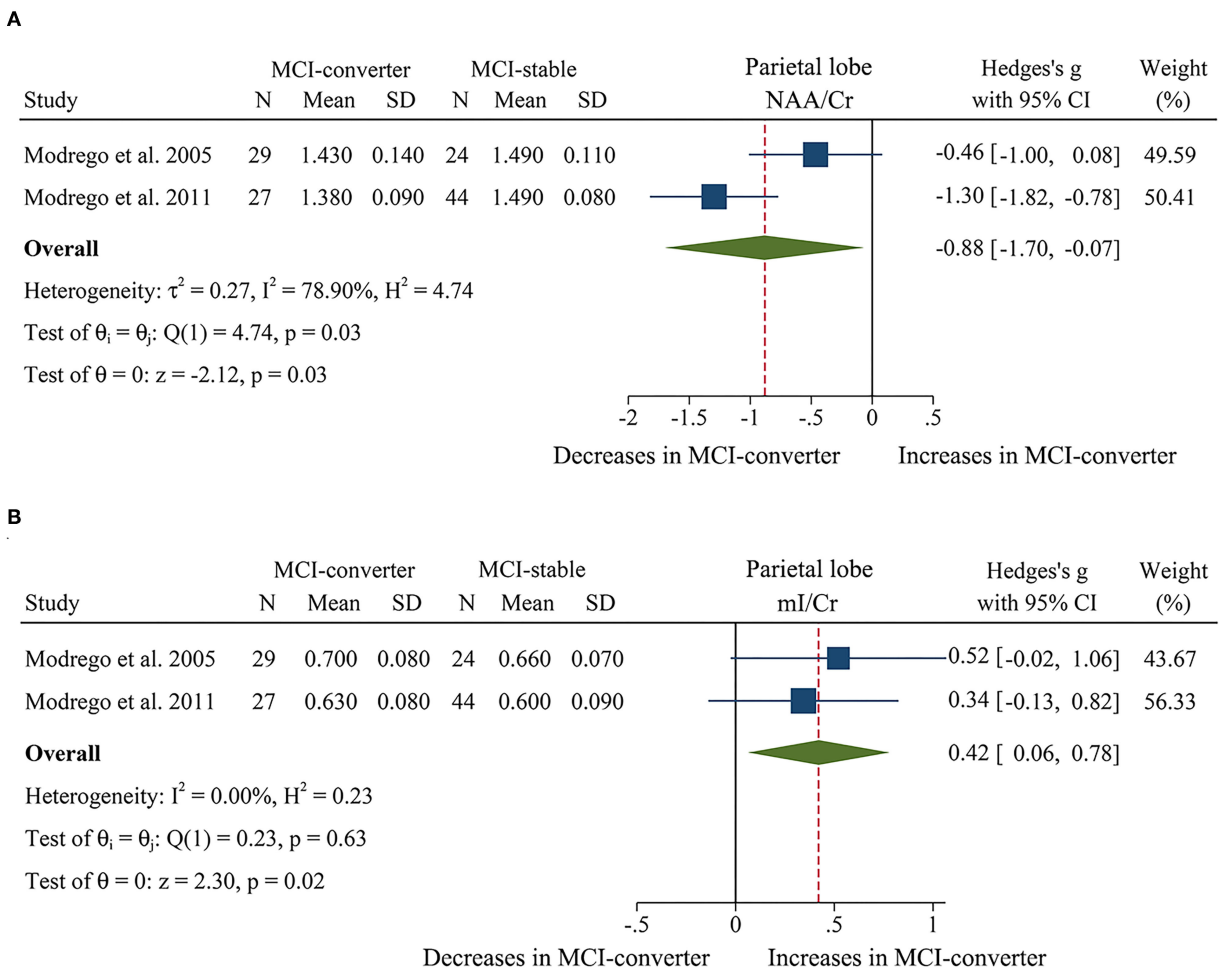
Forest plots show the change of NAA/Cr and mI/Cr in parietal lobe between MCI-converter and MCI-stable patients. **(A)** Data include 124 individuals from 2 studies for meta-analysis of NAA/Cr levels between MCI-converter and MCI-stable patients. **(B)** Data include 124 individuals from 2 studies for meta-analysis mI/Cr levels between MCI-converter and MCI-stable patients.

**Figure 9:**
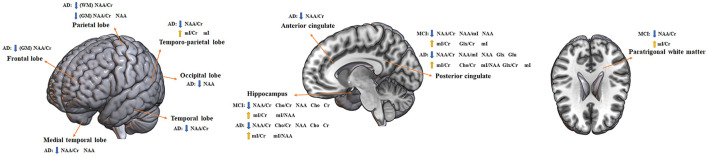
Altered metabolites in MCI and AD brain structures. AD, Alzheimer's disease; MCI, mild cognitive impairment; WM, white matter; GM, gray matter; NAA, N-acetyl aspartate; Cr, creatine; mI, myo-inositol; Cho, choline; Glx, glutamate + glutamine; Glu, glutamine. Directions: down, decrease; up, increase.

When comparing the metabolite ratios between 56 MCI-converter patients and 68 MCI-stable patients in the parietal lobe (Modrego et al., 2005, 2011), there were significant differences in two ratios between the two groups. The results demonstrated that NAA/Cr (SMD: −0.88 [95% CI: −1.70 to −0.07], *z* = −2.12, *P* < 0.05, Figures 8A, 9) was significantly higher than that in the MCI-converter patients, while the ratio of mI/Cr (SMD: 0.42 [95% CI: 0.06–0.78], *z* = 2.30, *P* < 0.05, Figures 8B, 9) was lower than that in the MCI-converter patients. Besides, the results revealed that there was no difference in Cho/Cr (SMD: 0.15 [95% CI: −0.21 to 0.50], *z* = 0.82, *P* > 0.1, Supplementary Table 1) and NAA/mI (SMD: −0.08 [95% CI: −0.92 to 0.76], *z* = −0.18, *P* > 0.1, Supplementary Table 1) between the two groups.

#### Metabolite Concentrations

We compared the concentrations of metabolites, extracting data from 96 AD patients and 102 healthy control subjects in 3 studies (Chao et al., 2005; Zhu et al., 2006; Siger et al., 2009). The results showed that the concentrations of NAA were significantly decreased in the parietal GM of AD patients (SMD: −0.95 [95% CI: −1.24 to −0.66], *z* = −6.36, *P* < 0.05, Supplementary Figure 5C and Figure 9).

### Meta-Analysis of the Occipital Lobe

There were 5 studies (Block et al., 2002; Azevedo et al., 2008; Watanabe et al., 2010; Graff-Radford et al., 2014; Marjańska et al., 2019) with a total sample size of 481 (195 AD patients and 286 healthy controls) included for meta-analysis to investigate the ratio of metabolites in the occipital lobe. Moreover, there were 3 more studies (Modrego et al., 2005, 2011; Seo et al., 2012) longitudinally tracking the metabolite differences between MCI-converter and MCI-stable patients.

#### Metabolite Ratios

Of the eligible studies, 3 (Block et al., 2002; Azevedo et al., 2008; Graff-Radford et al., 2014) reported data on metabolite ratios. These studies comprised 109 AD patients and 201 healthy controls. The results showed that there was a downward trend in NAA/Cr of AD patients (SMD: −0.22 [95% CI: −0.47 to 0.04], *z* = −1.69, *P* > 0.05, Supplementary Table 1), while there was no difference in Cho/Cr between the two groups (SMD: 0.22 [95% CI: −0.18 to 0.63], *z* = 1.08, *P* > 0.1, Supplementary Table 1).

Then, we identified 3 studies (Modrego et al., 2005, 2011; Seo et al., 2012) with a total sample size of 127 (63 MCI-converter and 74 MCI-stable patients) to compare the ratio in the occipital lobe The results demonstrated that NAA/Cr was significantly higher than that in the MCI-converter patients (SMD: −0.98 [95% CI: −1.98 to 0.02], *z* = −1.93, *P* > 0.05, Supplementary Table 1), while there were no differences in mI/Cr (SMD: −0.02 [95% CI: −0.37 to 0.34], *z* = −0.09, *P* > 0.1), Cho/Cr (SMD: −0.12 [95% CI: −0.45 to 0.22], *z* = −0.67, *P* > 0.1, Supplementary Table 1), and NAA/mI (SMD: −0.44 [95% CI: −1.44 to 0.56], *z* = −0.87, *P* > 0.1, Supplementary Table 1).

#### Metabolite Concentrations

Three studies (Azevedo et al., 2008; Watanabe et al., 2010; Marjańska et al., 2019) were extracted to compare the concentrations in 99 AD patients and 100 healthy controls. The results revealed that NAA concentrations were significantly decreased in the AD patients (SMD: −0.33 [95% CI: −0.62 to −0.05], *z* = −2.29, *P* < 0.05, Supplementary Figure 6A and Figure 9), while there were no differences in the concentrations of Cho (SMD: −0.11 [95% CI: −0.40 to 0.17], *z* = −0.80, *P* > 0.1, Supplementary Table 1), Cr (SMD: −0.21 [95% CI: −0.49 to 0.07], *z* = −1.45, *P* > 0.1, Supplementary Table 1), and mI (SMD: 1.09 [95%CI: −0.87 to 3.05], *z* = 1.09, *P* > 0.1, Supplementary Table 1).

### Meta-Analysis of Anterior Cingulate

Three studies (Lim et al., 2012; Guo et al., 2016; Huang et al., 2017) investigated the anterior cingulate including 66 MCI patients, 83 patients with AD, and 70 healthy control subjects. Specifically, 3 studies (Lim et al., 2012; Guo et al., 2016; Huang et al., 2017) performed a comparison in the changes between 66 MCI patients and 70 healthy control subjects, 3 studies (Lim et al., 2012; Guo et al., 2016; Huang et al., 2017) performed a comparison in the changes between 83 AD patients and 70 healthy control subjects, and 3 studies (Lim et al., 2012; Guo et al., 2016; Huang et al., 2017) were conducted to observe the differences of metabolites in 83 AD patients and 66 MCI patients.

#### Metabolite Ratios

First, we identified 3 studies (Lim et al., 2012; Guo et al., 2016; Huang et al., 2017) with a total sample size of 136 (66 MCI patients and 70 healthy controls) to compare the ratio of NAA/Cr in the anterior cingulate. The analysis showed that there was no difference between the two groups (SMD: −0.20 [95% CI: −0.68 to 0.29], *z* = −0.80, *P* > 0.1, Supplementary Table 1).

For the comparisons between 83 AD patients and 70 healthy controls (Lim et al., 2012; Guo et al., 2016; Huang et al., 2017), NAA/Cr was significantly decreased in the anterior cingulate of AD patients (SMD: −0.45 [95% CI: −0.77 to −0.13], *z* = −2.75, *P* < 0.05, Supplementary Figure 6B and Figure 9).

The comparison of 83 AD patients and 66 MCI patients in the ratio of NAA/Cr (Lim et al., 2012; Guo et al., 2016; Huang et al., 2017) revealed that there was no difference between the two groups (SMD: −0.25 [95% CI: −0.88 to 0.39], *z* = −1.10, *P* > 0.1, Supplementary Table 1).

### Meta-Analysis of the Temporo-Parietal Lobe

Four studies (Ernst et al., 1997; Fernández et al., 2005; Rami et al., 2007; Zhang et al., 2009) investigated the temporo-parietal lobe including 80 AD patients and 71 healthy control subjects to compare the metabolites between the two groups.

#### Metabolite Ratios

We compared the ratios of metabolites, extracting data from 157 AD patients and 110 healthy control subjects in 4 studies (Ernst et al., 1997; Fernández et al., 2005; Rami et al., 2007; Zhang et al., 2009). The results showed that two metabolites' ratios (NAA/Cr, mI/Cr) were significantly different in AD and healthy control subjects. NAA/Cr was significantly decreased in the temporo-parietal lobe of AD patients (SMD: −0.72 [95% CI: −1.36 to −0.07], *z* = −2.18, *P* < 0.05, Supplementary Figure 6C and Figure 9), while mI/Cr were significantly increased (SMD: 1.43 [95% CI: 0.60–2.27], *z* = 3.36, *P* < 0.05, Supplementary Figure 6D and Figure 9).

#### Metabolite Concentrations

Three studies (Ernst et al., 1997; Fernández et al., 2005; Rami et al., 2007) were extracted to compare the concentrations in 67 AD patients and 58 healthy controls. Specifically, mI was significantly increased in AD patients (SMD: 1.37 [95% CI: 0.26–2.48], *z* = 2.42, *P* < 0.05, Supplementary Figure 6E and Figure 9). There were no differences in NAA (SMD: −0.17 [95% CI: −0.51 to 0.18], *z* = −0.93, *P* > 0.1, Supplementary Table 1), Cho (SMD: −0.10 [95% CI: −0.44 to 0.25], *z* = −0.55, *P* > 0.1, Supplementary Table 1), and Cr (SMD: 0.51 [95% CI: −0.61 to 1.62], *z* = 0.89, *P* > 0.1, Supplementary Table 1) concentrations between the two groups.

### Meta-Analysis of the Frontal Region

Four studies (Parnetti et al., 1997; Chao et al., 2005; Zhu et al., 2006; Siger et al., 2009) with a total sample size of 218 (109 AD patients and 109 healthy controls) were included for meta-analysis to investigate the ratio of metabolites in the frontal region. Specifically, 3 studies (Parnetti et al., 1997; Zhu et al., 2006; Siger et al., 2009) compared the differences in metabolites between 61 AD patients and 61 healthy control subjects in the frontal WM, and 3 studies (Chao et al., 2005; Zhu et al., 2006; Siger et al., 2009) compared the differences in metabolites between 96 AD patients and 102 healthy control subjects in the frontal GM.

#### Metabolite Concentrations

We compared the concentrations of metabolites in the frontal WM, extracting data from 61 AD patients and 61 healthy control subjects in 3 studies (Parnetti et al., 1997; Zhu et al., 2006; Siger et al., 2009). The results showed that the concentration of mI has an upward trend in AD patients (SMD: 0.64 [95% CI: −0.06 to 1.34], *z* = 1.80, *P* > 0.05, Supplementary Table 1), and there was no significant difference in the concentrations of NAA between the two groups (SMD: −0.15 [95% CI: −0.50 to 0.21], *z* = −0.80, *P* > 0.1, Supplementary Table 1). Besides, 3 studies (Parnetti et al., 1997; Zhu et al., 2006; Siger et al., 2009) were included to investigate the concentration of NAA in the frontal GM and the analysis manifested a remarkable decrease with high heterogeneity (SMD: −0.37 [95% CI: −0.65 to −0.09], *z* = −2.63, *P* < 0.05, Supplementary Figure 6F and Figure 9).

### Meta-Analysis of Paratrigonal White Matter

Three studies (Catani et al., 2001; Metastasio et al., 2006; Yang et al., 2012) reported data from paratrigonal white matter including 89 MCI patients and 177 healthy control subjects to compare the metabolites between the two groups.

#### Metabolite Ratios

We compared the ratios of metabolites, extracting data from 89 MCI patients and 177 healthy control subjects in 3 studies (Catani et al., 2001; Metastasio et al., 2006; Yang et al., 2012). The results showed that two metabolites' ratios (NAA/Cr, mI/Cr) were significantly different between the two groups, and there was no significant difference in the ratio of Cho/Cr (SMD: 0.00 [95% CI: −0.26 to 0.25], *z* = −0.01, *P* > 0.1, Supplementary Table 1). Among them, NAA/Cr (SMD: −0.76 [95% CI: −1.02 to −0.49], *z* = −5.66, *P* < 0.05, Supplementary Figure 7A and Figure 9) was significantly decreased in paratrigonal white matter of MCI patients, while mI/Cr (SMD: 1.02 [95% CI: 0.20–1.84], *z* = 2.44, *P* < 0.05, Supplementary Figure 7B and Figure 9) was significantly increased.

## Discussion

To investigate the changes of neurochemicals estimated by ^1^H-MRS in brain regions with the progression of AD, we conducted a comprehensive meta-analysis including 63 studies with 3,271 subjects. The results showed that: (1) compared with MCI patients, the ratio of NAA/Cr in the hippocampus of AD patients decreased significantly; the ratios of NAA/Cr, NAA/mI, and the concentration of NAA in PC decreased significantly, whereas the ratios of mI/Cr and mI/NAA increased markedly. NAA and mI were considered as potential biomarkers for monitoring the progression from MCI to AD and early diagnosis of AD; (2) the metabolite difference of neurochemicals between MCI and AD was systematically analyzed and has found that the concentration of Glx in PC was different between MCI and AD patients, with an increase in AD but no changes in MCI groups. Therefore, Glx was crucial in differentiating MCI from AD, and was regarded as a potential marker to predict AD.

### Metabolic Changes of Neurochemicals During the Progression From MCI to AD

NAA is an important indicator of neuronal metabolism and plays a number of roles, which includes maintaining fluid balance in the brain, providing acetic acid salt for the synthesis of myelin in oligodendrocytes, and providing energy for the glutamylation of amino acid in neuronal mitochondria. Also, NAA is considered as a biomarker of neuronal function and density, as it can reflect the density and functional state of neurons and axons. Moreover, the concentration of NAA in the cortex can provide information about the growth of neurons. In addition, its concentration in white matter can reflect the development of axons. Due to NAA being located at the synaptic terminal, dendrites, and neuronal somata, its concentration may reflect the level of synapses and the ratio of NAA/Cr reflects the integrity of synapses (Onwordi et al., 2021). Our meta-analysis showed that during the pathological development from MCI to AD, the ratio of NAA/Cr in the hippocampus and PC, and the concentration of NAA in PC decreased dramatically. Meanwhile, the level of NAA/Cr in AD patients was decreased than that in the healthy subjects in the other brain lobes, such as the parietal lobe, the frontal lobe, the temporal lobe, and the temporo-parietal lobe. Previous studies showed that the hippocampus and PC were crucial brain regions that are differentially affected by neuropathological changes in AD patients (Silverman et al., 1997). Hippocampal atrophy is one of the pathological and radiological signs of AD, and the resting state functional magnetic resonance imaging and ^18^F-deoxyglucose PET demonstrated that hippocampal dysfunction is closely related to the cognitive impairment in AD patients (Yan et al., 2020). The PC plays a crucial role in the default mode network of the brain, and previous studies demonstrated that the functional connections between the PC and the hippocampus have also been weakened during cognitive impairment (Huang et al., 2002). Studies have reported that the atrophy of the hippocampus was closely related to the loss of neurons and the number of neurons had a close connection with NAA (Shiino et al., 2012). The results of this meta-analysis suggested that with the pathological development of AD, the neurons in the hippocampus and the PC were gradually damaged. And the formation of Aβ and NFT in the brain of AD patients may lead to the decrease of NAA and the gradual loss of synapses, which is consistent with the progress of cognitive dysfunction in AD. Similarly, there are studies showing that the sensitivity and specificity of NAA/Cr of PC in predicting the conversion of MCI to AD were 82% and 72%, respectively (Fayed et al., 2008). Consequently, NAA may be considered as a biomarker for monitoring the progression of MCI to AD.

mI is a good indicator of the proliferation of glial cells, as it is mainly expressed in glial cells. Previous studies have reported the increased levels of mI and mI/Cr in the PC were found in MCI and AD patients (Yang et al., 2012), which was consistent with our research results. In this meta-analysis, the results showed that in the pathological development from MCI to AD, in addition to the decrease in the ratios of NAA/Cr and the concentration of NAA in PC, there was also an increase in the ratios of mI/Cr and mI/NAA; and, compared with the HC, the mI concentration in the PC of AD and MCI patients was significantly increased. Meanwhile, the level of mI in MCI and AD patients was significantly higher than that in the healthy subjects in the other brain regions, such as the temporo-parietal lobe and PWM. The increase in mI concentration may be caused by the activation of astrocytes or microglia, which is related to the neuroinflammation process, and has been considered as one of the core pathological features of AD. In addition, the increased mI may affect the phosphorylation of membrane proteins or cause changes in phospholipid metabolism, affecting the formation of Aβ, and thus leading to the deposition of amyloid plaques. In addition, the increased deposition of Aβ also induced the formation of nutritionally impaired synapses, and the astrocytes wrapped and phagocytosed the diseased synapses to remove the aberrations in the synapses. However, with the development of the disease, the deposition intensified, and this pathological change promoted the increase of inflammatory response, which would disrupt the normal form of synapses (Gomez-Arboledas et al., 2018). Studies have found that the change in the ratio of mI/Cr in PC was closely related to the early decline of cerebrospinal fluid Aβ42, and the decrease in the level of CSF Aβ42 can be detected 10–20 years before the onset of cognitive impairment (Bateman et al., 2012). Studies have found in the brain of Down's syndrome and other dementia patients, the ratio of mI/Cr was also significantly increased before significant manifestations of cognitive dysfunction (Voevodskaya et al., 2016). Therefore, these results suggested that the change in the level of mI may precede the onset of cognitive impairment, which had the potential to be applied to early diagnosis of AD.

Moreover, it is worth noting that this study showed that the concentration of Cr was significantly lower in the hippocampus of AD and MCI patients than that of healthy people, but there was no significant change in other brain regions such as PC. Interestingly, it is generally believed that the concentration of Cr is basically constant and uniformly distributed throughout the brain and is not changed with age or various diseases. Therefore, the level of Cr is often used as a reference value to indicate the level of other neurochemical substances. Some studies have also found that the concentration of Cr was relatively reduced in the late stage of AD as well as subcortical ischemic vascular dementia, which may be due to the fact that Cr existed in neurons and glial cells at the same time, and was affected by the density of brain tissue (Shiino et al., 2012).

Studies have regarded the ratio of mI/NAA a standard method to determine the severity of AD, as the sensitivity and specificity of the ratio of mI/NAA in the diagnosis for AD patients were 83 and 98%, respectively, and was consistent with the MMSE score (Shiino et al., 2012). This meta-analysis found that the ratio of mI/NAA increased during the progression from MCI to AD in PC, and the same result was also observed in the hippocampus. But since only 3 studies were included and a large heterogeneity was observed, this result should be interpreted cautiously. In addition, there was no meta-analysis results in the procession from MCI to AD in the hippocampus. Therefore, the change of mI/NAA is consistent in the progression from MCI to AD, but whether it can be used as markers in early diagnosis of AD is still questionable.

### Different Metabolic Changes of Neurochemicals Between MCI and AD

The findings with respect to changes in the levels of Cho and Glx in MCI and AD were less consistent. As discussed above, the ratio of Cho/Cr was significantly higher in the PC of AD patients than that of HC, but there was a downward trend in MCI subjects. In addition, the concentration of Cho was found to be raised in the PC of AD patients compared with healthy controls, and no differences were seen in the MCI subjects. In contrast, the concentration of Cho was found to be reduced in the hippocampus of AD patients and MCI patients compared with healthy controls. It had been reported that a cholinergic lesion emerged as early as the MCI state and primarily in the presynaptic membrane, which may affect the long-term potentiation (Nordberg and Winblad, 1986). Cho plays an important role in the formation of cell membranes, and the change of concentration directly reflects the synthesis and degradation of membranes. Cho could be converted into acetylcholine (AchE) by choline acetyltransferase (ChAT) to play a neuroregulatory role, and the Cho signal may be closely related to the activity of ChAT (Klein, 2000). A clinical autopsy study found increased ChAT activity in the hippocampus of patients with MCI, which could explain that the decrease of Cho in the MCI stage is due to the increased activity of ChAT and the utilization of more choline substrates, thus resisting the damaging effect of cholinergic neurons (Ikonomovic et al., 2003). This compensatory activity may increase with the progression of the disease. Meanwhile, neuronal death will lead to an increase in membrane turnover, which will increase the ratio of Cho/Cr in AD patients. Recent investigations reported that the increase in the level of Cho in PC of AD patients may be the result of cell membrane rupture providing free Cho, which was in response to a decrease in the release of acetylcholine from cholinergic neurons in the brain of AD patients (Watanabe et al., 2010). In frontotemporal dementia and dementia with Lewy bodies, the increase in the ratio of Cho/Cr in the PC can also be detected. Interestingly, there was no significant change in the early stage of AD disease. In MCI patients, the concentration of Cho in PC remained basically the same with healthy controls, while the ratio of Cho/Cr had an upward trend, which may be due to the gradual aggravation of cholinergic neuron damage with the progression of the disease. Currently, cholinergic inhibitors such as donepezil, rivastigmine, and galanthamine are clinically used to treat AD. A meta-analysis showed that these drugs had modest but clinically significant overall benefits in stabilizing cognition, function, behavior, and overall clinical changes (Tan et al., 2014). Therefore, the change of Cho may reflect the severity of AD and was considered as a potential target for early detection and interventions.

In recent years, more research has focused on the change of Glx in patients with MCI and AD. Glx is a class of excitatory amino acid, including glutamate (Glu) and glutamine (Gln) (Bleich et al., 2003). In the brain, Glu and Gln are in dynamic equilibrium, and they can maintain and regulate synaptic information transmission through mutual transformation. In addition, Glu plays a crucial role in mitochondrial metabolism, neurotransmission of pyramidal cells, cerebral cortex function, and glutamate/GABA-glutamine cycle. And Glu-mediated synaptic transmission is critical for brain functions. However, excessive and continuous excitatory glutamatergic stimulation can lead to the death of neurons (Fayed et al., 2011). Interestingly, our results showed that compared with HC, the concentrations of Glx and Glu in PC of AD patients were lower, while the ratio of Glx/Cr was relatively higher, and the ratio of Glx/Cr in the PC of MCI patients was higher, while the concentration of Glu had a downward trend. In animal experiments, it was also found that the Glu/Cr was decreased in AD model mice (Liang et al., 2017). Studies have reported that the Aβ can induce several changes in nerve cells including the loss of neuronal viability and synaptic activity, leading to the reduction in glutamate levels. Meanwhile, the decrease of Glu content will affect the A-amino-3-hydroxy-5-methyl-4-isoxazolepropionic acid receptors (AMPARs), which play a key role in synaptic function and cognition. In addition, this decrease in AMPARs may be the reason for the loss of synaptic and the decrease of cognitive function in AD (Liu et al., 2010). Consequently, Glx/Cr and Glu may be seen as signs of cognitive deterioration in AD.

### Limitations

Several limitations to the current meta-analysis should be pointed out. First, the number of longitudinal studies to investigate the changes of metabolites between MCI-converter and MCI-stable patients was limited, so the sample size for analysis was relatively small. Therefore, more longitudinal studies are required to observe and explain the metabolite changes during the progress of MCI to AD. In addition, a significant effect of heterogeneity was found in many studies, and we were temporarily unable to do any moderating analysis to detect systematic influence on heterogeneity. Additionally, the detection results of MRS are affected by multiple parameters such as TR, TE, and ROI. This meta-analysis did not unify these parameters, which may lead to heterogeneity and affect the results.

## Conclusion

In conclusion, the main findings of our meta-analysis revealed robust metabolite changes in the PC and the hippocampus during the development from MCI to AD, especially the levels of NAA and mI show high accuracy in the discrimination between healthy controls, MCI, and AD, but were also able to predict the possible progression from MCI to AD.

## Data Availability Statement

The raw data supporting the conclusions of this article will be made available by the authors, without undue reservation.

## Author Contributions

SL, SH, and HLiu designed the study. SL and HLin revised the manuscript. HLiu, DZ, and HLin wrote the initial manuscript. HLiu, DZ, LZ, YZ, XY, and ZL collected the data and undertook the statistical analysis. SL and QZ critically reviewed and modified the paper. All authors contributed to the article and approved the submitted version.

## Funding

This work was supported by the grants from the National Natural Science Foundation of China (82004440), Fujian Provincial Health Technology Project (2019-1-65), and Scientific Research Foundation for the High-Level Talents funded by Fujian University of Traditional Chinese Medicine (X2019002-talents and X2019014-talents).

## Conflict of Interest

The authors declare that the research was conducted in the absence of any commercial or financial relationships that could be construed as a potential conflict of interest.

## Publisher's Note

All claims expressed in this article are solely those of the authors and do not necessarily represent those of their affiliated organizations, or those of the publisher, the editors and the reviewers. Any product that may be evaluated in this article, or claim that may be made by its manufacturer, is not guaranteed or endorsed by the publisher.
