# Supplementary material for: Meta-Analysis of Neurochemical Changes Estimated via Magnetic Resonance Spectroscopy in Mild Cognitive Impairment and Alzheimer's Disease
Source: Front Aging Neurosci. 2021 Oct 22;13:738971. doi: 10.3389/fnagi.2021.738971 (PMC8569809; doi:10.3389/fnagi.2021.738971)

**Supplementary Table 1** results of meta-analysis

| Region | Subjects | Metabolites | No. of Studies | $I^2$ | $H^2$ | Z | P | SMD | 95%CI |
|---|---|---|---|---|---|---|---|---|---|
| Hippocampus | MCI Vs HC | mI | 4 | 58.99% | 2.44 | 1.08 | 0.28 | 0.23 | -0.19 to 0.65 |
| | | Glx/Cr | 3 | 86.62% | 7.47 | -1.44 | 0.15 | -0.76 | -1.81 to 0.28 |
| | AD Vs MCI | mI/Cr | 4 | 29.18% | 1.41 | 1.92 | 0.06 | 0.25 | -0.01 to 0.50 |
| | MCI-converter Vs MCI-stable | Cho/Cr | 2 | 0.00% | 0.03 | -1.91 | 0.06 | -0.47 | -0.94 to 0.01 |
| | | NAA/Cr | 2 | 17.31% | 1.21 | -0.72 | 0.47 | -0.17 | -0.65 to 0.30 |
| PC | MCI Vs HC | mI/NAA | 3 | 76.15% | 4.19 | 0.04 | 0.97 | 0.02 | -0.79 to 0.82 |
| | | Cho/Cr | 17 | 89.01% | 9.10 | 1.96 | 0.05 | 0.34 | -0.00 to 0.69 |
| | | NAA/Cho | 4 | 58.33% | 2.40 | -1.80 | 0.07 | -0.35 | -0.72 to 0.03 |
| | AD Vs HC | Cr | 4 | 73.18% | 3.73 | -1.71 | 0.09 | -0.37 | -0.80 to 0.05 |
| | | Cho | 6 | 50.97% | 2.04 | 1.81 | 0.07 | 0.23 | -0.02 to 0.48 |
| | AD Vs MCI | Cho/Cr | 9 | 56.41% | 2.29 | 0.79 | 0.43 | 0.10 | -0.15 to 0.35 |
| | | mI | 4 | 0.00% | 0.37 | -0.69 | 0.49 | -0.07 | -0.28 to 0.13 |
| | | Cho | 3 | 76.46% | 4.25 | -0.17 | 0.87 | -0.05 | -0.57 to 0.48 |
| | MCI-converter Vs MCI-stable | NAA/Cr | 2 | 0.00% | 0.07 | 0.68 | 0.50 | 0.17 | -0.33 to 0.67 |
| | | Cho/Cr | 2 | 0.00% | 0.64 | 0.44 | 0.66 | 0.11 | -0.39 to 0.61 |
| Temporal lobe | MCI Vs HC | NAA/Cr | 3 | 0.00% | 0.30 | -0.81 | 0.42 | -0.12 | -0.40 to 0.17 |
| | AD Vs HC | Cho/Cr | 3 | 19.57% | 1.24 | 1.91 | 0.06 | 0.35 | -0.01 to 0.71 |
| | | mI/Cr | 4 | 0.00% | 0.50 | -1.87 | 0.06 | -0.27 | -0.57 to 0.01 |
| Parietal lobe | MCI Vs HC | NAA/Cr | 3 | 0.00% | 0.41 | 0.16 | 0.88 | 0.02 | -0.2 to 0.24 |
| | MCI-converter Vs MCI-stable | Cho/Cr | 2 | 35.30% | 1.55 | 0.82 | 0.41 | 0.15 | -0.21 to 0.50 |
| | | NAA/mI | 2 | 81.64% | 5.45 | -0.18 | 0.85 | -0.08 | -0.92 to 0.76 |
| Occipital lobe | AD Vs HC | NAA/Cr | 3 | 0.00% | 0.47 | -1.69 | 0.09 | -0.22 | -0.47 to 0.04 |
| | | Cho/Cr | 3 | 54.88% | 2.22 | 1.08 | 0.28 | 0.22 | -0.18 to 0.63 |

| Region | Subjects | Metabolites | No. of Studies | $I^2$ | $H^2$ | Z | P | SMD | 95%CI |
|---|---|---|---|---|---|---|---|---|---|
| Occipital lobe | AD Vs HC | Cho | 3 | 43.21% | 1.76 | -0.80 | 0.42 | -0.11 | -0.40 to 0.17 |
| | | Cr | 3 | 28.62% | 1.40 | -1.45 | 0.15 | -0.21 | -0.49 to 0.07 |
| | | mI | 3 | 96.34% | 27.29 | 1.09 | 0.28 | 1.09 | -0.87 to 3.05 |
| | MCI-converter Vs MCI-stable | NAA/Cr | 3 | 83.86 | 6.20 | -1.93 | 0.05 | -0.98 | -1.98 to 0.02 |
| | | mI/Cr | 2 | 0.00% | 0.68 | -0.09 | 0.93 | -0.02 | -0.37 to 0.34 |
| | | NAA/mI | 2 | 86.41% | 7.36 | -0.87 | 0.38 | -0.44 | -1.44 to 0.56 |
| AC | MCI Vs HC | NAA/Cr | 3 | 50.97% | 2.04 | -0.80 | 0.42 | -0.20 | -0.68 to 0.29 |
| | AD Vs MCI | NAA/Cr | 3 | 73.20% | 3.73 | -1.10 | 0.45 | -0.25 | -0.88 to 0.39 |
| temporo-parietal lobe | AD Vs HC | NAA | 3 | 32.36% | 1.48 | -0.93 | 0.35 | -0.17 | -0.51 to 0.18 |
| | | Cho | 3 | 88.49% | 8.69 | 0.89 | 0.37 | 0.51 | -0.61 to 1.62 |
| | | Cr | 3 | 0.00% | 0.56 | -0.55 | 0.59 | -0.10 | -0.44 to 0.25 |
| Frontal WM | AD Vs HC | mI | 3 | 68.55% | 3.18 | 1.80 | 0.07% | 0.64 | -0.06 to 1.34 |
| | | NAA | 3 | 47.43% | 1.90 | -0.80 | 0.42% | -0.15 | -0.50 to 0.21 |
| PWM | MCI Vs HC | Cho/Cr | 3 | 0.00% | 0.54 | -0.01 | 0.99% | 0.00 | -0.26 to 0.25 |

HC: healthy controls, AD: Alzheimer's disease, MCI: Mild Cognitive Impairment, SMD: standardized mean difference, PC: posterior cingulate cortex, PWM: paratrigonal white matter, AC: anterior cingulate cortex, WM: white matter, NAA: N-Acetyl Aspartate, mI: myo-inositol, Cho: Choline, Cr: Creatine, Glx: glutamate + glutamine.

**Supplementary Figure S1** Forest plots show the change of neurochemicals in hippocampus between MCI patients and HC subjects. **(A)** Data include 429 individuals from 7 studies for meta-analysis of Cho/Cr levels between MCI and HC. **(B)** Data include 465 individuals from 8 studies for meta-analysis mI/Cr levels between MCI and HC. **(C)** Data include 85 individuals from 3 studies for meta-analysis of mI/NAA levels between MCI and HC. **(D)** Data include 317 individuals from 4 studies for meta-analysis of NAA levels between MCI and HC. **(E)** Data include 317 individuals from 4 studies for meta-analysis of Cho levels between MCI and HC. **(F)** Data include 317 individuals from 4 studies for meta-analysis of Cr levels between MCI and HC.

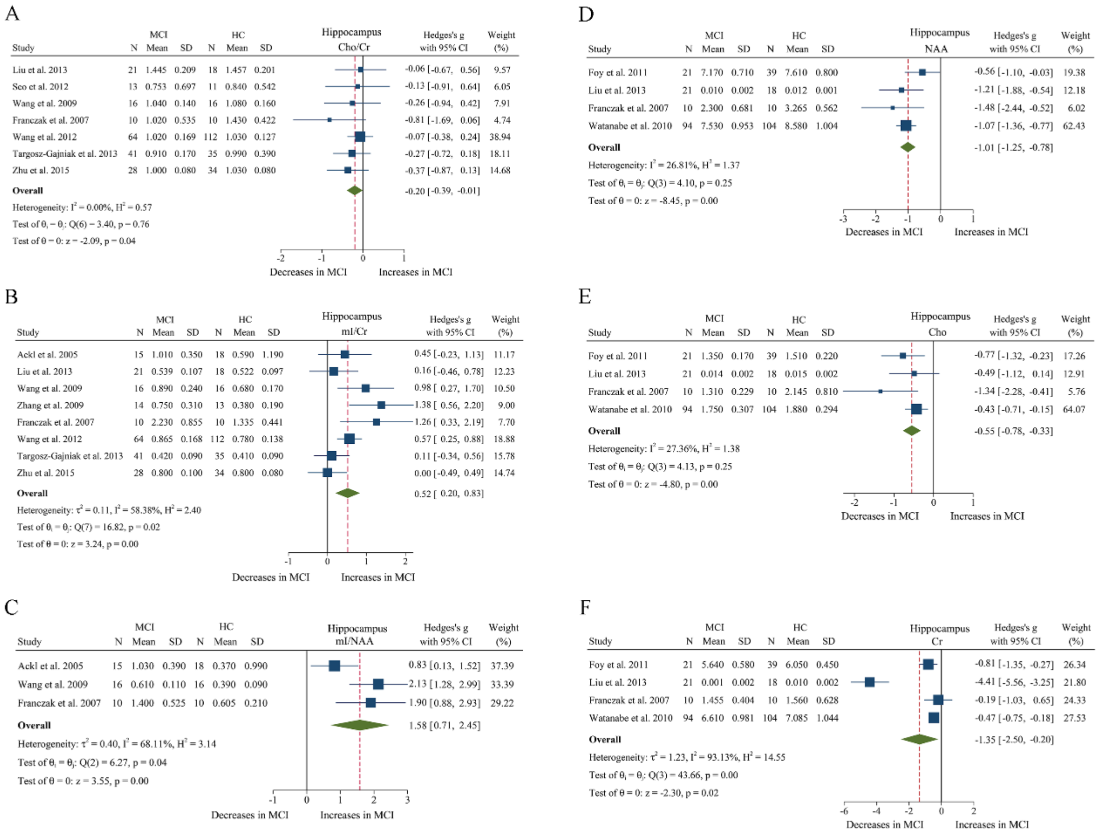

**Supplementary Figure S2** Forest plots show the change of neurochemicals in hippocampus between AD patients and HC subjects. **(A)** Data include 604 individuals from 4 studies for meta-analysis of Cho/Cr levels between AD and HC. **(B)** Data include 584 individuals from 5 studies for meta-analysis mI/Cr levels between AD and HC. (**C)** Data include 352 individuals from 3 studies for meta-analysis of mI/NAA levels between AD and HC. (**D**) Data include 829 individuals from 4 studies for meta-analysis of NAA levels between AD and HC. **(E)** Data include 609 individuals from 3 studies for meta-analysis of Cho levels between AD and HC. **(F)** Data include 609 individuals from 3 studies for meta-analysis of Cr levels between AD and HC.

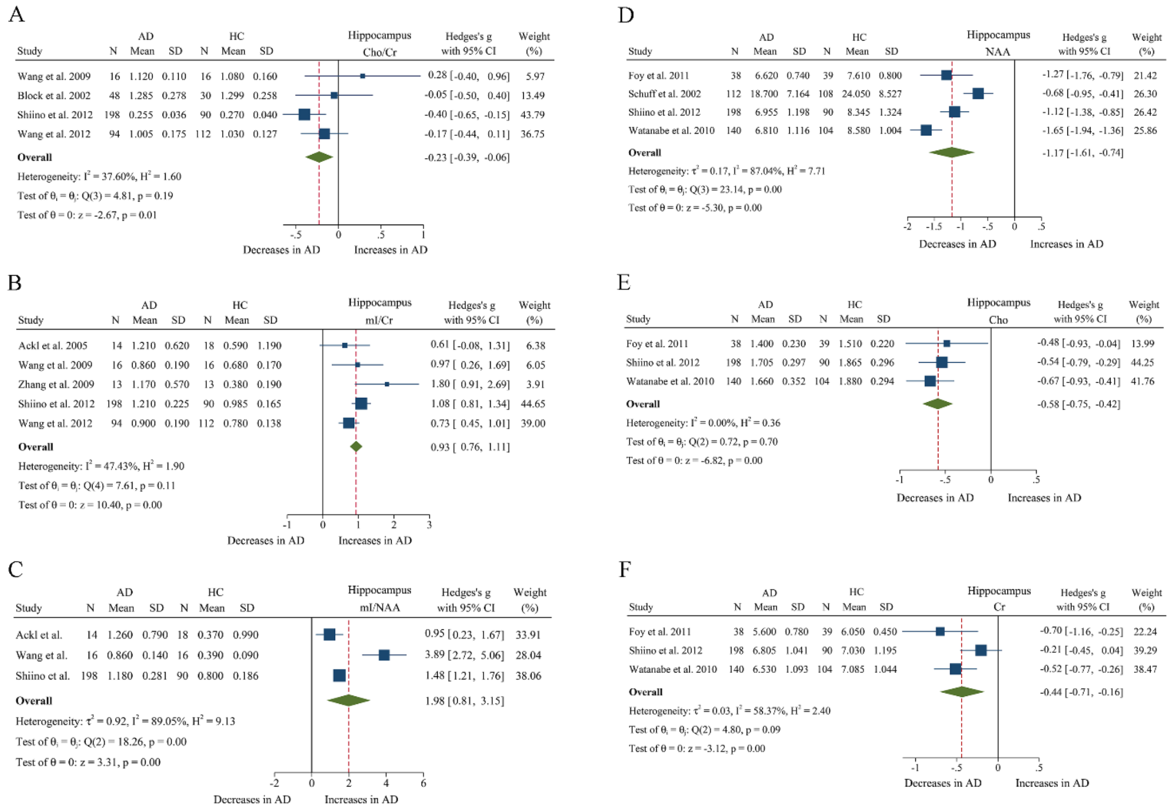

**Supplementary Figure S3** Forest plots show the changes of NAA/Cr and NAA in medial temporal lobe between AD patients and HC subjects. **(A)** Data include 333 individuals from 4 studies for meta-analysis of NAA/Cr levels between AD and HC. **(B)** Data include 509 individuals from 4 studies for meta-analysis NAA levels between AD and HC.

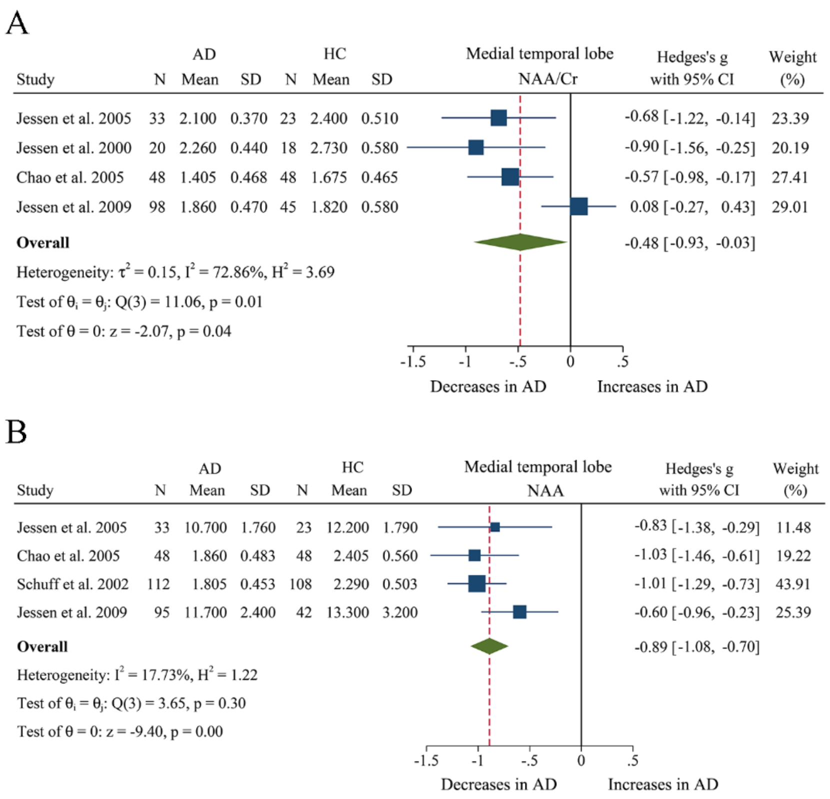

**Supplementary Figure S4** Forest plots show the change of neurochemicals in posterior cingulate and temporal lobe between MCI, AD patients and HC subjects. **(A)** Data include 279 individuals from 4 studies for meta-analysis of mI/NAA levels between AD and HC. **(B)** Data include 1159 individuals from 12 studies for meta-analysis Cho/Cr levels between AD and HC. **(C)** Data include 120 individuals from 3 studies for meta-analysis of mI/NAA levels between AD and MCI. **(D)** Data include 841 individuals from 9 studies for meta-analysis of mI levels between MCI and HC. **(E)** Data include 662 individuals from 6 studies for meta-analysis of mI levels between AD and HC. **(F)** Data include 267 individuals from 5 studies for meta-analysis of NAA/Cr levels in temporal lobe between AD and HC.

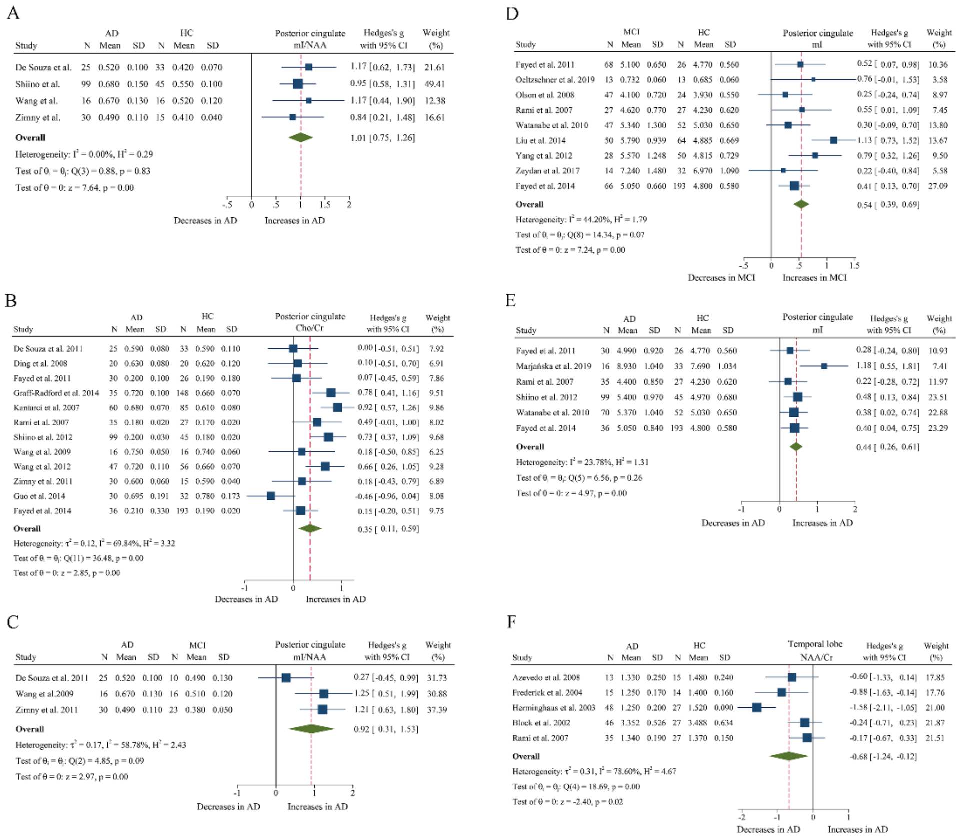

**Supplementary Figure S5** Forest plots show the change of NAA/Cr and NAA in parietal lobe between AD patients and HC subjects. **(A)** Data include 151 individuals from 3 studies for meta-analysis of NAA/Cr levels in the Parietal WM between AD and HC. **(B)** Data include 151 individuals from 3 studies for meta-analysis NAA/Cr levels in Parietal GM between AD and HC. **(C)** Data include 198 individuals from 3 studies for meta-analysis of NAA levels in the Parietal GM between AD and HC.

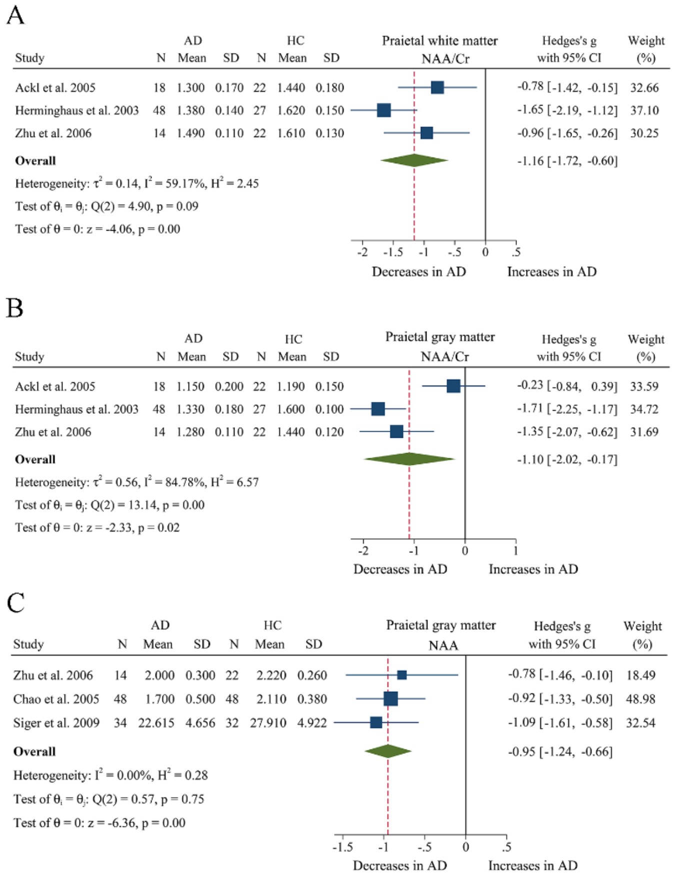

**Supplementary Figure S6** Forest plots show the change of NAA and NAA/Cr in occipital lobe, anterior cingulate, temporo-parietal lobe and frontal region between AD patients and HC subjects. **(A)** Data include 199 individuals from 3 studies for meta-analysis of NAA levels in occipital lobe between AD and HC. **(B)** Data include 136 individuals from 3 studies for meta-analysis NAA/Cr levels in the anterior cingulate between AD and HC. (C) Data include 151 individuals from 4 studies for meta-analysis of NAA/Cr levels in temporo-parietal lobe between AD and HC. **(D)** Data include 151 individuals from 4 studies for meta-analysis of mI/Cr levels in temporo-parietal lobe between AD and HC. **(E)** Data include 125 individuals from 3 studies for meta-analysis of mI levels in temporo-parietal lobe between AD and HC. **(F)** Data include 198 individuals from 3 studies for meta-analysis of NAA levels in frontal GM between AD and HC.

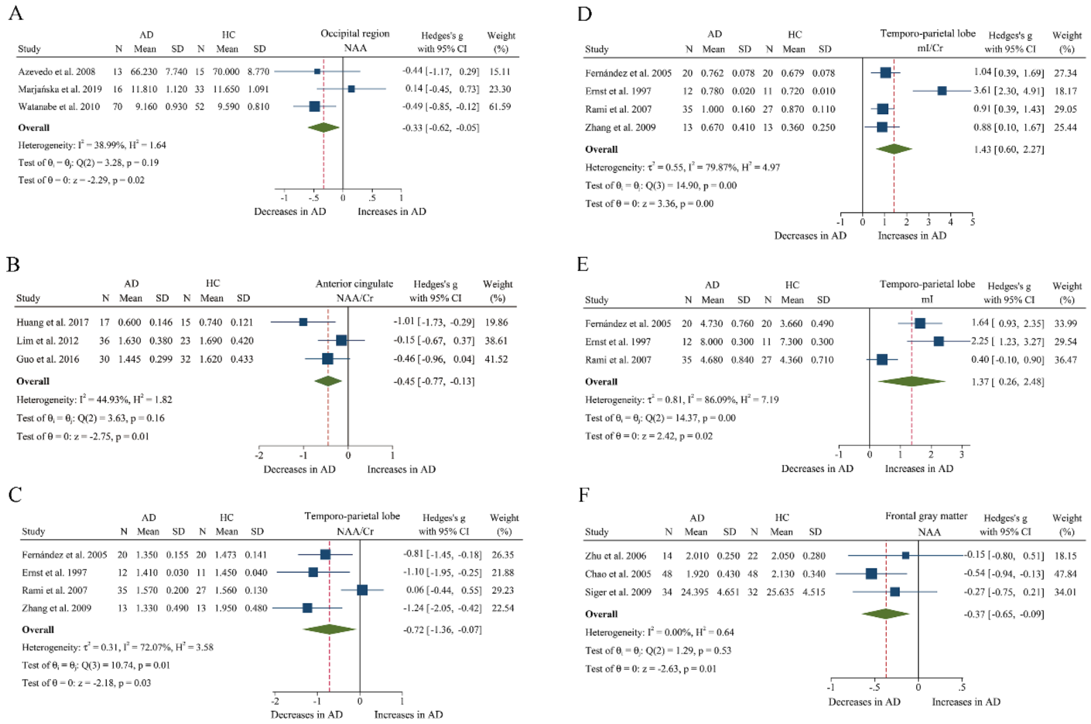

**Supplementary Figure S7** Forest plots show the change of NAA/Cr and mI/Cr in paratrigonal white matter lobe between MCI patients and HC subjects. **(A)** Data include 266 individuals from 3 studies for meta-analysis of NAA/Cr levels between MCI and HC. **(B)** Data include 266 individuals from 3 studies for meta-analysis mI/Cr levels in anterior cingulate cortex between AD and HC.

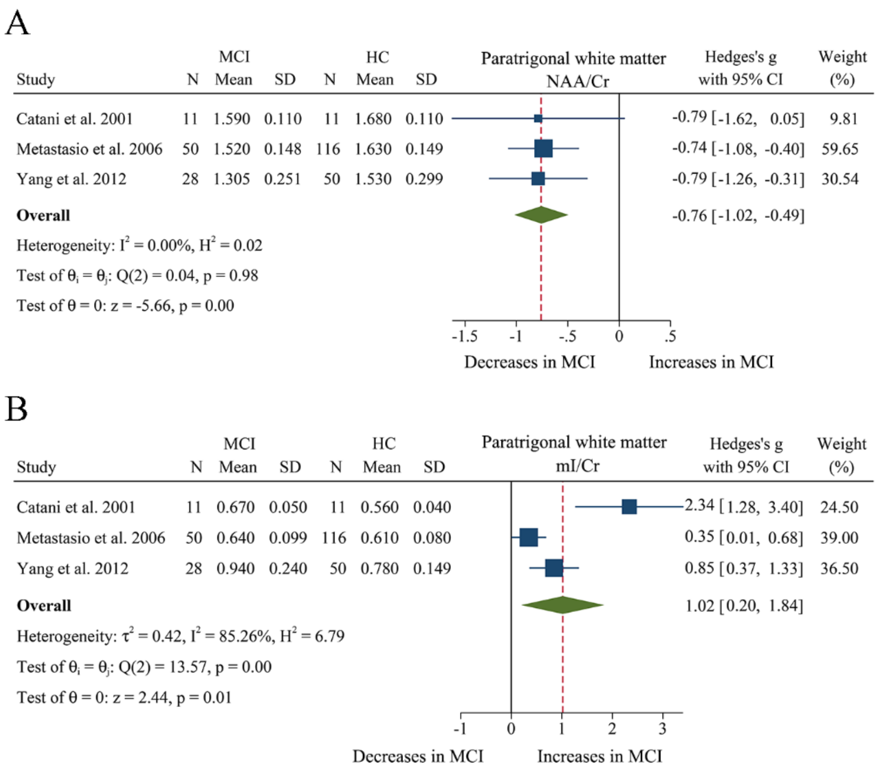

Supplement: Supplementary file 1 [file Data_Sheet_1.pdf]
